# Deep learning unmasks the ECG signature of Brugada syndrome

**DOI:** 10.1093/pnasnexus/pgad327

**Published:** 2023-10-13

**Authors:** Luke Melo, Giuseppe Ciconte, Ashton Christy, Gabriele Vicedomini, Luigi Anastasia, Carlo Pappone, Edward Grant

**Affiliations:** Department of Chemistry, University of British Columbia, Vancouver, BC V6T 1Z1, Canada; Arrhythmia and Electrophysiology Center, IRCCS Policlinico San Donato, Milan 20097, Italy; Department of Chemistry, University of British Columbia, Vancouver, BC V6T 1Z1, Canada; Arrhythmia and Electrophysiology Center, IRCCS Policlinico San Donato, Milan 20097, Italy; Stem Cell Laboratory for Tissue Engineering, Università Vita-Salute San Raffaele, Milan 20132, Italy; Arrhythmia and Electrophysiology Center, IRCCS Policlinico San Donato, Milan 20097, Italy; Department of Cardiology, Università Vita-Salute San Raffaele, Milan 20132, Italy; Department of Chemistry, University of British Columbia, Vancouver, BC V6T 1Z1, Canada

**Keywords:** artificial intelligence, Brugada Syndrome, deep neural network, electrocardiogram, Sudden Cardiac Death

## Abstract

One in 10 cases of sudden cardiac death strikes without warning as the result of an inherited arrhythmic cardiomyopathy, such as Brugada Syndrome (BrS). Normal physiological variations often obscure visible signs of this and related life-threatening channelopathies in conventional electrocardiograms (ECGs). Sodium channel blockers can reveal previously hidden diagnostic ECG features, however, their use carries the risk of life-threatening proarrhythmic side effects. The absence of a nonintrusive test places a grossly underestimated fraction of the population at risk of SCD. Here, we present a machine-learning algorithm that extracts, aligns, and classifies ECG waveforms for the presence of BrS. This protocol, which succeeds without the use of a sodium channel blocker (88.4% accuracy, 0.934 AUC in validation), can aid clinicians in identifying the presence of this potentially life-threatening heart disease.

**Significance Statement**

Sudden cardiac death (SCD) persists as a significant public health problem, claiming millions of lives every year, including many young and otherwise healthy individuals. Among patients without evident structural heart disease, SCD often occurs as the first clinical manifestation of an inherited disorder, such as Brugada Syndrome (BrS). Unfortunately, such patients seldom show overt signs of disease. The present study demonstrates that a deep neural network can detect the signature of BrS in an ECG, even in the frequent case when these signs are undetectable by the human eye. This tool, when applied to routine clinical ECGs, will alert clinicians to potentially life-threatening conditions that would otherwise go undiagnosed.

## Introduction

Sudden cardiac death (SCD) kills more than 4.2 million people worldwide every year (1). Among young and otherwise healthy individuals, SCD often appears without warning as the first clinical manifestation of an inherited arrhythmic disease (2–4), such as Brugada Syndrome (BrS) (5–8). Two-thirds of patients afflicted with BrS show no symptoms of cardiovascular disease, and SCD often serves as its first clinical manifestation (5).

The hidden and dynamic nature of this disease underlines the need for an improved diagnostic test (9). The incidence of confirmed BrS varies by country (5); but, the difficulty of diagnosis coupled with the complex genetic architecture (10, 11) and inheritability of this disease suggests that its true prevalence is unknown. Most affected patients present a normal ECG, and nearly half of BrS-afflicted individuals who survive a cardiac arrest fail to show a diagnostic type 1 pattern in a routine ECG (12). Both factors severely hinder the recognition of this lethal disease.

**Fig. 1.**
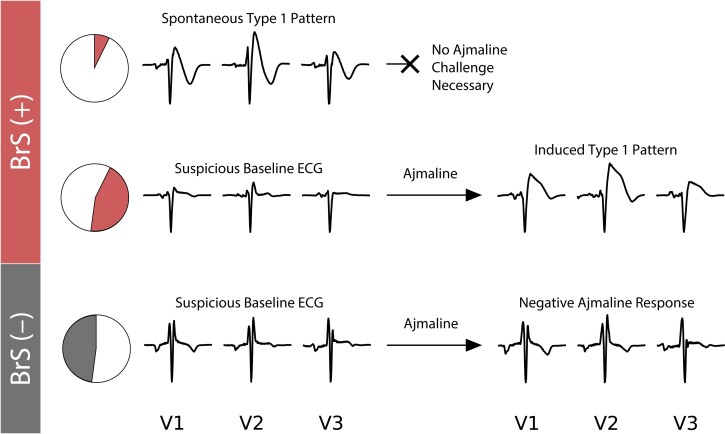
Representative heartbeats recorded for V1, V2, and V3 in (*left*) baseline electrocardiograms of three typical patients diagnosed as spontaneous type 1 BrS(+) (*top*), BrS(+) (*middle*), and BrS(−) (*bottom*). Electrocardiograms are recorded during ajmaline administration for patients not presenting a spontaneous type 1 pattern (*right*). Note the ST-elevation feature signifying BrS in the type 1 ECG traces. In our study, only 17% of BrS(+) patients exhibit a spontaneous type 1 pattern, while the remaining 83% of patients required an ajmaline challenge to reveal a hidden type 1 pattern.

Where permitted, cardiologists may administer one of several sodium channel blockers (SCB) to unmask the BrS type 1 pattern (5, 13, 14) as demonstrated in Fig. 1. However, these drugs can cause life-threatening proarrhythmic effects, which limit their use to those individuals with a known family history of SCD (15, 16). And ajmaline, the most effective diagnostic SCB, is unavailable for clinical use in many countries. including the United States of America and Japan. Thus, the development of an easily applied, nonintrusive BrS diagnostic test remains an important and pressing objective.

The interpretation of an electrocardiogram for medical diagnosis requires the application of expert knowledge in a full consideration of the many interconnected factors that influence ECG waveform patterns. This central problem of learning and retention lends itself well to an approach that classifies on the basis of a deep neural network (DNN), tied to a very large library of digital waveform standards (17). An extensive body of effort has applied artificial intelligence with the processing power of modern computers to recognize diagnostic features of gross rhythm disorders such as atrial fibrillation, and certain common cardiomyopathies for which large digital ECG databases exist (18–30). However, thus far, machine learning has yet to establish digital signatures for more subtle, but no less-threatening conditions such as BrS, often harbored by asymptomatic individuals who present apparently normal ECGs. The difficulty of assembling a large-scale digitized dataset of expertly diagnosed ECGs presents a significant challenge to progress (31).

Here, we describe the development of a robust noninvasive method to diagnose the electrophysiological signature of BrS from the digital analysis of a routine clinical electrocardiogram. This work differs substantially from the many other machine-learning approaches to ECG classification, particularly those that target BrS with only a capacity to identify affected individuals who present a visible type 1 pattern in their baseline ECG (32–35).

Rather than surveying a continuous ECG for abnormalities, much like a medical professional might perform a visual assessment, our approach reduces a standard multi-lead ECG trace to a set of high-quality representative single heartbeats, one for each lead, and then classifies upon this basis. This preprocessing strategy better isolates the persistent baseline function of the heart’s electrical conduction system by overcoming transient uncorrelated beat-to-beat variance present in the raw traces. This dimensionality reduction helps the neural network recognize the persistent ECG features that most consistently encode for BrS.

## Methods

### Study population

**Fig. 2.**
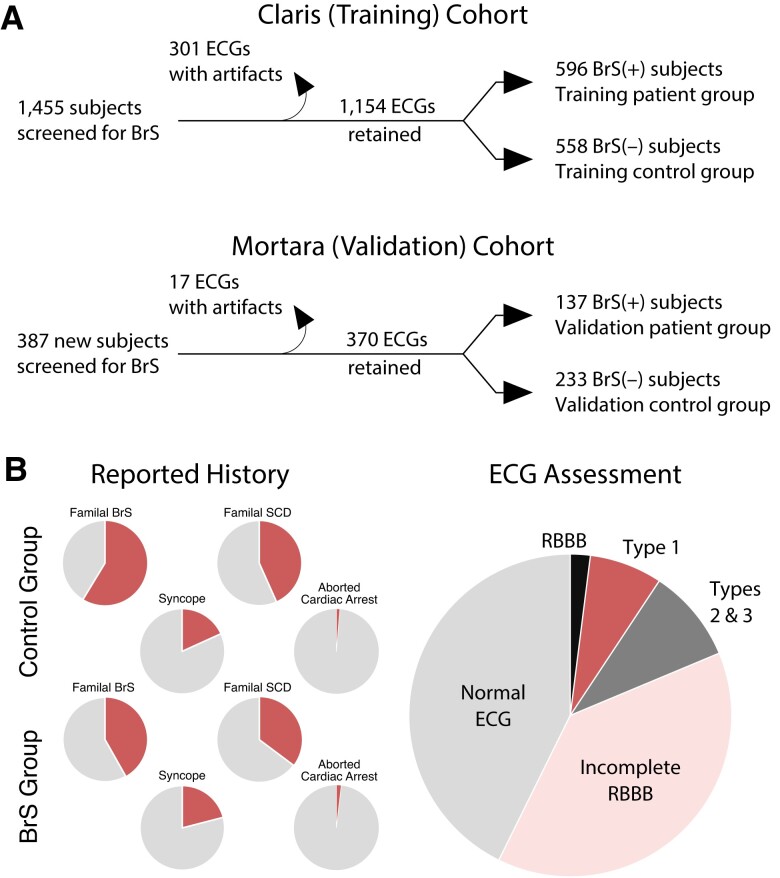
(A) Flowcharts illustrating the organization of electrocardiograms into one cohort for the purposes of training and validating DNN models, and into a second cohort for independent validation. (B) Distributions of patient clinical statistics, including medical and family history in the Claris training cohort. RBBB refers to right bundle branch block. Complete details can be found in the Supplementary material.

The present study draws upon an existing database of 1,455 consecutive patients undergoing electrophysiological evaluation for the diagnosis of BrS at the Arrhythmia Department of IRCCS Policlinico San Donato (San Donato Milanese, Milano, Italy), beginning in April 2015 (Fig. 2 and Table S1).

A second set of ECGs, collected in a separate clinic beginning in March 2020, serves as a second independent validation cohort, consisting of 405 consecutive new patients in the Brugada screening program (Table S2). The data collected for each patient include a physical examination, complete personal and family medical histories, and a complete set of digital ECG traces.

These populations each contain two overarching classification groups. Patients in the Brugada Group BrS(+) present evidence of a spontaneous or drug-induced type 1 BrS ECG pattern. The Control Group BrS(−) is comprised of patients, all of whom presented a negative sodium channel blocker test, defined as an ECG that does not convert to yield a type 1 pattern after a pharmacological provocation test (ajmaline injection at the maximal dose of 1 mg/kg, according to the current consensus statement (5)). Although we obtained ECGs for eligible patients during ajmaline administration to confirm BrS diagnoses, we trained and tested our classification algorithms exclusively on baseline ECGs, recorded without ajmaline.

The study patients presented as candidates for ajmaline infusion owing to diagnostic indicators of risk, associated with the possibility of BrS for at least one of the following reasons: (i) Family history of SCD or family history of BrS, (ii) unexplained syncope, (iii) nondiagnostic Brugada pattern or suspicious ECG, (iv) aborted cardiac arrest, (v) unexplained ventricular arrhythmias. The exclusion criteria for the ajmaline test included the presence of a spontaneous type 1 pattern (n=106;7.3%), an established diagnosis of an overt structural cardiomyopathy, and severe conduction disturbances (e.g. left or right bundle branch block with QRS>140ms). During the study period, a small number of patients (n=53;3.5%) refused to undergo the ajmaline test. This retrospective study subscribed to the plan described in a protocol approved by the Ethical Committee of the I.R.C.C.S. Policlinico San Donato. All participants provided informed consent.

### Acquisition and storage of ECG traces

All ECG records were digitally acquired and stored in the Arrhythmology Department digital repository at the IRCCS Policlinico San Donato. The principal calibration and validation effort relied upon measurements made using a set of Workmate Claris instruments (Abbott Laboratories, Abbott Park, IL, USA). The hospital outpatient clinic afforded ECGs for the supplemental validation cohort recorded using a Mortara ELI 350 system (Mortara Instrument, Inc., Milwaukee, WI, USA).

Both systems were configured to simultaneously acquire and digitize two limb leads (L1, L2) and six precordial leads (V1 through V6), and automatically reconstruct the 12-lead ECG. The Claris system records the ECG with a sampling frequency of 2,000 Hz and a resolution of 5 μV, while the Mortara system records at a sampling frequency of 1,000 Hz and a resolution of 1 μV. All ECG records were collected using a customized high precordial lead placement optimized for the detection of the BrS ECG pattern. The six precordial leads were placed at the left and right parasternal positions in the second (V1 II ICS, or V1; V2 II ICS, or V2), third (V1 III ICS, or V3; V2 III ICS, or V4), and fourth intercostal spaces (V1 IV ICS, or V5; V2 IV ICS, or V6).

All patients exhibited stable sinus rhythm during baseline ECG acquisition without premature ectopies. All baseline ECG records were analyzed by three trained and independent cardiologists, who each provided an initial index classification: (i) normal (i.e. no evident abnormalities resembling a BrS pattern), (ii) spontaneous type 1 BrS pattern, and (iii) suspicious BrS pattern (i.e. ECG abnormalities including but not limited to complete or incomplete right bundle branch block, type 2, or type 3 morphology).

Routine diagnosis calls for the acquisition of a baseline ECG, followed by one recorded prior to and during an ajmaline challenge for all cases, except for patients whose baseline ECG exhibited a spontaneous type 1 BrS pattern. Approximately one minute of an ECG recording prior to ajmaline administration was recorded, anonymized, and exported as a set of text files from the hospital database. Additional patient clinical characteristics, including age, sex, personal history of syncope and cardiac arrest, and family history of sudden death and BrS diagnosis, were also logged in the export files as additional factors of variation. This information was archived in an SQL database. ECG traces recorded on the Mortara system were collected for a new set of patients upon admission to the hospital and reserved for independent validation. ECG traces recorded from both systems are subject to the same ECG trace processing, outlined below.

### ECG trace processing

**Fig. 3.**
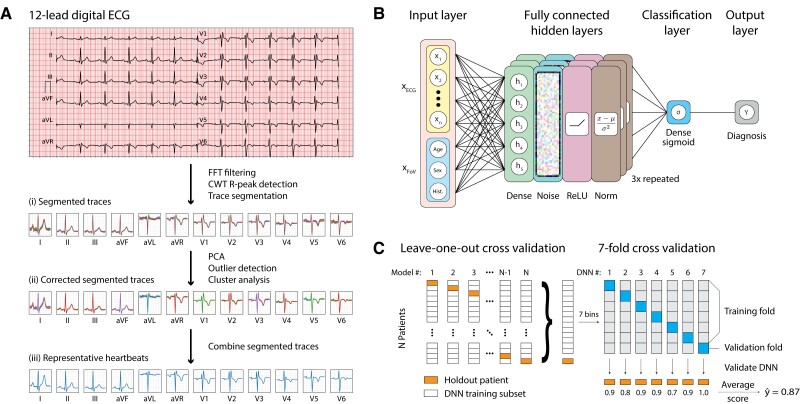
(A) Flowchart illustrating the ECG data processing workflow from trains of heartbeats as monitored by each ECG lead, to segmentation, outlier discrimination, and averaging to form a set of single representative beats. (B) Structure of the neural network used for training the Brugada syndrome classifier including input, hidden, and output layers. The hidden layer unit includes a fully connected layer followed by Gaussian noise, ReLU activation, and batch normalization. The hidden layer unit is repeated three times, followed by a fully-connected output layer with sigmoid activation. (C) Data partitioning strategy for training a neural network. The overall data partitioning scheme implements leave-one-out cross-validation (LOOCV) to assess the performance of independent neural networks constructed for every patient. For each patient, a 7-fold cross-validation separates the dataset into seven training and validation subsets, each tested on the holdout patient. This analysis yields an average score and standard deviation for each patient across the 7-fold DNNs.

We reduce the pulse train in each lead to a single de-noised and representative heartbeat. This procedure, which is detailed in the Supplementary material, combined with down-sampling to 200 Hz, yields a much sparser representation (Fig. 3a). Averaged, single-lead heartbeats, concatenated end-to-end, forms a fused, one-dimensional representation of the entire electrocardiogram for each patient. The results presented in this study classify on the basis of a 9-lead ECG (all leads excluding the unipolar limb leads aVF, aVR, and aVL). In the Supplementary material, we additionally explore individual and other lead combinations in detail.

The results reported here stem from the analysis of retrospective clinical databases. The ECGs in these databases were not recorded for the purpose of digitally training a machine-learning algorithm, but rather to enable a clinician to assess visually for the presence of diagnostic features. Consequently, we found it necessary to screen for measurement artifacts and deemed some recordings unsuitable for multivariate analysis. We performed this screening manually prior to any application of machine learning. As diagrammed in Fig. 2a, 1,455 unique patients yielded a set of 1,154 high-quality ECGs. A total of 370 of 405 ECGs met acceptance standards in the Mortara validation cohort.

### Neural networks

The representative heartbeats for each patient, along with the attendant factors of variation populate a design matrix **X${}_{ECG}$**. We define a disease-state vector **Y** by assigning 0 or 1 to every ECG, representing Brugada-negative and-positive, respectively, determined by the presence or the absence of a type 1 ECG pattern, confirmed where necessary by a positive response to ajmaline.

We train a deep neural network (DNN), consuming the elements of the design matrix **X${}_{ECG}$**. supervised by the disease state **Y**, to optimize the parameters of a classification function *F*(**X${}_{ECG}$**) =  **Y** (Fig. 3b). Details about the training, optimization, and validation procedures for our neural networks can be found in the Supplementary material. All computer codes developed for ECG processing, neural network training, and statistical analysis are written in-house using Python 3.9 and Tensorflow 2.

### Independent validation of DNNs

Two distinct tests confirm the validity with which the DNN models developed in this work classify newly encountered ECGs for BrS. A large-scale process of leave-one-out cross-validation (LOOCV) (Fig. 3c) independently gauges the degree to which ensemble learning correctly classifies each and every individual patient in the training dataset.

In a procedure repeated for all *N* patients in the database, we withhold one patient in turn and use the N−1 other patients to train a distinct set of seven DNN models that are blind to the withheld patient. We employ ensemble learning for each N−1 training subset to minimize sampling bias and overfitting. Consequently, a unique set of seven DNN models trained on exclusive N−1 populations subsampled by 7-fold cross-validation independently classify each patient.

By design, the corresponding set of seven independent DNN models assigns a set of seven numbers between 0 and 1 to every withheld ECG, which we term the DNN score, or $\hat{y}$, which could be interpreted as a probability of positive diagnosis. We compute the final DNN score for each validated patient as the average of the scores produced by the corresponding seven exclusive DNNs. We further test the utility of a DNN model trained by all the Claris patients by deploying it to classify a second independent cohort of validation data containing 370 patients (Mortara dataset).

### Interpreting statistical results

We gauge the validation success of the DNN models by means of the areas under curve (AUC) of receiver-operating characteristics (ROC) plots (36). DNN scores close to 0 or 1 indicate high classification certainty for the absence or the presence of a BrS pattern, respectively, while scores close to the decision threshold of 0.5 point to ECGs that yield a low classification certainty. The decision threshold of 0.5 is determined using Youden’s *J* statistic (37, 38).

To assess our results, we compare continuous variables using a Student *t*-test and binomial variables with $\chi^{2}$ or Fisher’s exact test, as appropriate. We compute the AUC of ROC curves of DNN performance, determining confidence intervals by DeLong’s method (36), and recognizing statistical significance for p<0.05. Further details on statistical analyses and methods for minimizing overfitting are described in the Supplementary material.

## Results

### Independent validation within the claris training dataset

**Fig. 4.**
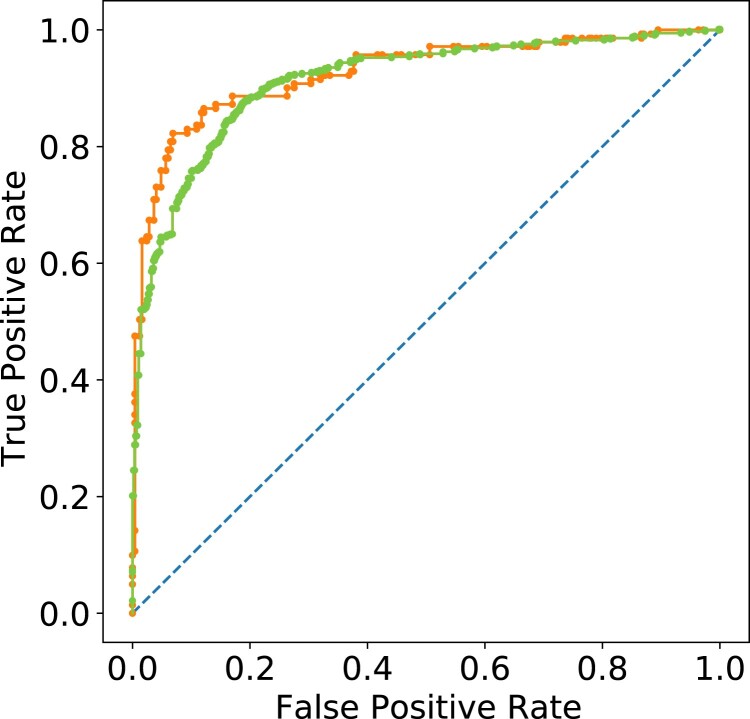
Receiver–operator characteristic curve for the Claris (training, green, plotted foreground) and Mortara (independent validation, orange, plotted foreground) cohorts.

The DNN, applied in LOOCV within the Claris (training) dataset, correctly predicts the ajmaline response for 84.0% (969 of 1,154) of patients, with an AUC of 0.912±0.017 (Fig. 4), a sensitivity of 85.4%, and a specificity of 82.4% (Tables 1, S1 and S5). Furthermore, the DNN classifies patients showing a spontaneous type 1 pattern with an accuracy of 100% (103 of 103).

**Table 1.** Classification metrics of the trained neural networks on independent holdout cases in the claris (training) and mortara (independent validation) cohorts.

Cohort	Classifier	Sensitivity	Specificity	PPV	NPV	Accuracy	AUC	*p*-value
*Claris (Training)*	DNN	85.4%	82.4%	83.9%	84.1%	84.0%	0.912±0.017	0.039
		(509 of 596)	(460 of 558)	(509 of 607)	(460 of 547)	(969 of 1,154)		
*Mortara (Validation)*	DNN	79.6%	93.6%	87.9%	88.6%	88.4%	0.934±0.027	–
		(109 of 137)	(218 of 233)	(109 of 124)	(218 of 246)	(327 of 370)		
	M.D. 1	71.5%	73.0%	60.9%	81.3%	72.4%	0.722±0.047	4.65E−08
		(98 of 137)	(170 of 233)	(98 of 161)	(170 of 209)	(268 of 370)		
	M.D. 2	55.5%	60.9%	45.5%	70.0%	58.9%	0.582±0.052	9.41E−20
		(76 of 137)	(142 of 233)	(76 of 167)	(142 of 203)	(218 of 370)		

For the Claris cohort (n=1,154), comprising 9-lead fused representative ECG traces, we perform independent validation of each case using 7-fold leave-one-out cross-validation (LOOCV). For the Mortara cohort (n=370), we report the results of the trained neural networks on independent holdout cases. *p*-values are calculated by a two-sided $\chi^{2}$ test between the DNN and Mortara accuracy scores. Delong’s method yields uncertainties for AUC-ROC values at a 95% confidence interval.

We determine that the 9-lead fused representative ECG trace yields the best classification performance by comparing AUC values for various lead combinations (Table S4). The incorporation of clinical factors of variation such as age, gender, and family history of cardiac events did not significantly change the DNN performance (Table S6). In a thorough exploration of classification strategies, we observed superior performance of the training dataset DNN to other popular supervised learning techniques such as a convolutional neural network (CNN) (Tables S8 and S9), decision trees, Naive Bayes, support vector machines (SVM), and k-nearest neighbors (KNN), among others (Table S12).

### Cross-platform independent validation

We performed a second independent validation of a seven-DNN ensemble trained on the basis of the 1,154 patients in the Claris (training) cohort by applying this model to classify for BrS in a new prospectively-studied cohort. Routine examinations at the IRCCS Policlinico San Donato outpatient clinic using several available Mortara ELI 350 instruments afforded a supplemental set of 370 ECGs, which served to define a separate, independent validation cohort. Table S3 details the composition and clinical characteristics of this cohort.

The DNN model, entirely calibrated and validated using data from the Claris platform, correctly identifies BrS(+) patients in the independent Mortara cohort with an accuracy of 85.1% (327 of 370), an AUC of 0.934±0.027 (See Fig. 4), a sensitivity of 79.6%, and a specificity of 93.6% (Table 1). The 9-lead classification metrics of this cross-platform independent validation compare with those of the DNN applied to the Claris cohort training data (Table 1). Additionally, the DNN correctly classified 100% of the spontaneous BrS type 1 patients. Table S8 further details the accuracy with which the DNN classifies the Mortara cohort.

### Comparative manual assessment of the mortara cohort

As a gauge of the present machine learning approach against standard clinical practice, we compiled the diagnoses of two clinicians at the IRCCS Policlinico San Donato, who visually inspected and classified the ECG records in the Mortara cohort, without knowledge of the accompanying ajmaline-confirmed BrS diagnoses. Table 1 summarizes the results. An expert cardiologist with more than 20 years of experience in clinical arrhythmology and electrophysiology (M.D. 1) classified the Mortara cohort ECGs with an accuracy of 72.4%, a sensitivity of 71.5%, and a specificity of 73.0%. Table S10 provides details. A resident clinician in training (M.D. 2) performed the same classification with an accuracy of 58.9%, a sensitivity of 55.5%, and a specificity of 60.9%. See Table S11. Most tellingly perhaps, AUC values for MD1 and MD2, 0.722±0.047 and 0.582±0.052, compare with 0.934±0.027 found in validation of the DNN by the Motara cohort.

### DNN feature importance

**Fig. 5.**
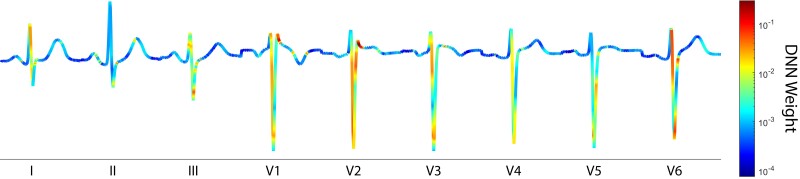
DNN feature importance shown on a 9-lead fused ECG trace, representative of the DNN training data. The colormap displays the magnitude of the weights applied by the DNN when predicting ajmaline response. The weights range from 0.0001 to 0.3021; higher–weighted regions have a greater influence on the DNN model.

Figure 5 shows a heatmap indicating the importance of ECG features as perceived by the DNN. The type 1 BrS pattern is characterized by a “coved-type” ST segment, while the type 2 pattern is characterized by a “saddle-back” shape with an ST elevation of ≥ 1mm in leads V1 to V3. As such, a neural network classification model that seeks only the type 1 or 2 patterns would likely place a significant weight on the ST-segment of leads V1 to V3. However, while our DNN model does assign some importance to these regions, it Classifies ECGs on the basis of a complex multivariate relation among waveform amplitudes, including a nonlinear combination of the ST-segments from V1 and V2, and the QRS complexes in leads I, III, V1, V2, and V6. We did not provide our DNN model with any input weights, nor did we label any regions of the ECG as important. Thus, the regions of the fused representative ECG indicated in Fig. 5 detail a positive response to the SCB challenge *ab initio*. Importantly, note that the DNN model assigns virtually no weight to the eight junctions at which we have concatenated the representative beats from each lead. This DNN indifference affirms the robustness of our preprocessing approach.

## Discussion

The present study demonstrates a neural network model that learns to recognize the signature of Brugada Syndrome hidden in a conventional ECG in the absence of any proarrhythmic drug challenge. These results establish that an appropriately trained DNN model responds to subtle ECG abnormalities that are undetectable to the human eye. The algorithm correctly classifies a majority of ECGs by predicting the response of a patient to an ajmaline challenge without the need for other clinical information. Despite the relatively limited size of this database, the neural network assesses apparently normal ECGs with an AUC of 0.912±0.017 (accuracy of 84.0%) and 0.934±0.027 (accuracy of 88.4%) in the training and validation datasets respectively.

To date, when evaluating a patient at an arrhythmia clinic, the clinician must decide whether to administer a sodium channel blocker in an effort to unmask the BrS phenotype (5, 13, 14, 39, 40). This drug challenge exposes the patient to the possibility of a life-threatening arrhythmia; but, a decision not to challenge risks overlooking a patient with hidden BrS. At present, owing to the possibility of life-threatening side effects, only patients with a family history of SCD or a suspicious ECG undergo a drug challenge.

Thus, many people who show no visible sign of BrS may well remain undiagnosed (9). The identification of BrS is particularly limited in countries, including the United States of America and Japan, where ajmaline is generally unavailable, and the SCB challenge is limited to less effective agents such as procainamide and flecainide. Moreover, several conditions expressly contraindicate this diagnostic approach. For example, a fatal ventricular arrhythmia calls for the assessment of family members for BrS; but, clinicians may hesitate to administer ajmaline to children. This limitation may leave many young carriers of BrS undiagnosed. These drugs are also contraindicated in cases of pregnancy, liver diseases, and advanced cardiac conduction disturbances.

Sodium channel blockers have negative inotropic properties that preclude use for patients suffering from cardiomyopathies, particularly those associated with mutations in the SCN5A gene and other conditions overlapping with BrS, such as hypertrophic cardiomyopathy (HCM) and arrhythmogenic right ventricular cardiomyopathy (ARVC) (41–44). Dynamic effects of these drugs vary a great deal from individual to individual, and this clouds the significance of any particular test result. The direct hepatotoxic effect of these agents further excludes their diagnostic applicability as a screening tool.

Thus, significant considerations limit the diagnostic application of SCB drugs compared with an instrumental measure of heart function, such as an electrocardiogram, which does not rely upon the action of an agonist. While work still remains to validate machine-learning classification algorithms for patients with accompanying conditions not considered in the present study, the accuracy demonstrated thus far, approaching that normally achieved in SCB tests, offers a potential of great benefit as a safe first step in screening a wider population.

To this purpose, several studies have reported the usefulness of some ECG parameters in the discrimination of BrS patterns (45–49). However, intra- and inter-observer variability in specific measurements limit the power of univariate pattern-recognition methods. More generally, the ability to identify ECG predictors of BrS depends on a clinician’s level of clinical experience. Very recently, a convolutional neural network approach showed success in recognizing the characteristic ST elevation of a type 1 BrS pattern (33). However, only a small fraction of BrS(+) individuals spontaneously present this plainly visible ECG feature.

Indeed, in the present cohort, roughly 90% of patients showed a near-normal or merely suspicious ECG waveform, challenging to evaluate even by experienced cardiologists. Thus, the development of this advanced DNN-based ECG interpretation algorithm promises to aid in the diagnosis of BrS by providing a discerning and objective tool to assist the clinician prior to the possible use of potentially harmful SCB challenges. This tool will add considerable insight when clinicians, in keeping with current guidelines, combine the results of an ECG with a full scope of other clinical data to establish a final diagnosis of BrS (5). The Supplementary material offers additional details concerning classification success upon manual versus DNN analysis with reference to patient demographics and clinical characteristics.

The sensitivity and specificity of our DNN model compare favorably with other diagnostic tests used in cardiology, such as exercise electrocardiography to diagnose coronary artery disease (50), and exceed other medical screening tests, such as the BNP immunoassay for heart failure (AUC 0.60–0.70) (51), the Papanicolaou smear for cervical cancer (AUC 0.70) (52), and the CHA2DS2-VASc Score for stroke risk (AUC 0.57–0.72) (53). Moreover, the relatively high negative predictive value (NPV) of the DNN classification will lend reassurance in decisions to forego the invasive and risky step of an ajmaline challenge. The current findings will translate to a clinical application that informs clinicians who are considering ordering an SCB challenge for suspected Brugada Syndrome.

Of 1,455 patients in the present cohort, 7% presented an ECG with a spontaneous type 1 pattern. Using standard clinical protocols, a conventional BrS screening program would have found only these patients. Among asymptomatic individuals in this study, we diagnosed more than 49% as having BrS. Most of these patients had no idea that they carried this condition. They were referred for screening only because of the sudden and unexplained death of a relative, reinforcing the idea that the actual prevalence of BrS is likely underestimated.

Remarkably, the DNN model correctly classifies every patient who presented a spontaneous type 1 ECG. This marker signals a heightened danger of cardiac arrest (5, 54, 55). But, nearly half of BrS patients who survive cardiac arrest do not show a spontaneous type 1 pattern, underlining the critical need to clinically identify patients that harbor a hidden disease signature and face a substantial probability of SCD (12). In young adults, the first clinical manifestation of BrS is often atrial fibrillation (56). This suggests that BrS may represent a complex and diffuse phenotype, sharing genetic mutations with various supraventricular arrhythmias (43, 57, 58), as well as with other cardiomyopathies, such as HCM (44), ARVC (10) and early repolarization syndrome (59).

This broader relevance points to an additional reach of noninvasive BrS screening as a means to reduce the risk of life-threatening arrhythmias and sudden death. This is particularly relevant for patients who present a nondiagnostic ECG pattern that may mask a clinically aggressive phenotype requiring further cardiac investigation. The classification success of the DNN suggests that machine learning will enable clinicians to identify these patients even from an apparently normal ECG.

Furthermore, as plainly evident in Fig. 4, a completely independent cross-platform dataset validates the DNN with virtually the same accuracy (AUC 0.93 vs 0.9) as LOOCV applied to the training dataset. This not only proves again that the algorithm generalizes well to classify previously unseen data, but also to successfully classify ECG data acquired on different machines. A larger ECG dataset and multiparametric diagnostic assessment, performed by combining ECG machine learning and genetic information (60), could provide better accuracy, and advance substantially in diminishing the devastating impact of a family of lethal diseases by screening the general population. Additionally, next-generation electrocardiographs and new implantable or wearable devices could support operating systems that incorporate artificial intelligence built upon this DNN model for output processing (61).

A DNN algorithm for assisting the diagnosis of BrS offers several advantages compared with current practices: (i) reduction of test-related adverse events, such as life-threatening arrhythmias and ajmaline-induced liver injury, (ii) reduction of the cost compared with an SCB test, which must be performed in a hospital setting, and (iii) identification of family members at risk of BrS in centers with limited access to drug testing or no access to ajmaline.

### Interpreting DNN feature importance

Understanding where a DNN places the greatest importance in the input data is crucial for interpreting the decisions a model makes (see Fig. 5). By identifying which ECG features the DNN weights most heavily in its classification model, we can understand which aspects of the ECG signal play the greatest role in identifying the BrS phenotype. This information can help guide the selection of new data for training, improve the interpretability of the model, and explain why the model might fail to correctly identify the BrS phenotype in some cases.

We observe that the DNN’s interpretation largely aligns with the cardiologists’ approach, as it considers not only ST elevation, but also other aspects of the ECG, including QRS interval, QT interval, J point depression, and the presence of an incomplete right bundle branch block. This convergence is significant, as it illustrates how the DNN learns to recognize important ECG features in a completely unbiased data-driven approach, supervised solely on a patient’s response to the ajmaline challenge.

DNN multivariate classification evaluates ECG data by means of thousands of nonlinear decision-making pathways, allowing it to identify patterns and features that escape notice in conventional approaches. This enables the DNN model to recognize subtle covariance of diagnostic significance. We believe that feature importance patterns established by our DNN approach could help refine and expand the understanding of Brugada Syndrome, potentially leading to the development of more robust diagnostic criteria.

The substantial data preprocessing method described here reduces a raw, noisy ECG trace to yield a representative heartbeat as a single, averaged complete cardiac cycle (750 ms in duration) centered near the R peak. The high signal-to-noise representative heartbeat yields a persistent, reproducible waveform for classification by the DNN. Our segmentation strategy overcomes uncorrelated beat-to-beat variance in the raw traces. This transformation aids the DNN in recognizing persistent ECG morphology that consistently encodes for BrS while ignoring transient abnormalities.

## Conclusion

The present work demonstrates an ECG screening algorithm for BrS that competes with the sodium-channel-blocker challenge while presenting the clear advantage of no life-threatening side effects. This mode of computer-assisted analysis can be applied to digital ECG traces from all current electrocardiographs. The broad application of this methodology will enable clinicians to test widely for this deadly disease. We foresee that this machine learning approach to ECG analysis will extend beyond the diagnosis of BrS to broader risk stratification and mitigation of SCD.

## Supplementary Material

pgad327_Supplementary_Data

## Data Availability

Summary demographic and diagnostic data are included in the manuscript and Supplementary material. Anonymized data created for the study are available in a persistent repository: Melo, Luke (2023). Deep learning unmasks the ECG signature of Brugada Syndrome [Dataset]. Dryad. https://doi.org/10.5061/dryad.s1rn8pkd9.

## References

[1] Zeppenfeld K , *et al*. 2022. 2022 ESC guidelines for the management of patients with ventricular arrhythmias and the prevention of sudden cardiac death: developed by the task force for the management of patients with ventricular arrhythmias and the prevention of sudden cardiac death of the European Society of Cardiology (ESC) endorsed by the Association for European Paediatric and Congenital Cardiology (AEPC). Eur Heart J. 43(40):3997–4126. 36017572 10.1093/eurheartj/ehac262

[2] Hayashi M, Shimizu W, Albert CM. 2015. The spectrum of epidemiology underlying sudden cardiac death. Circ Res. 116(12):1887–1906. 26044246 10.1161/CIRCRESAHA.116.304521 PMC4929621

[3] Priori SG , *et al*. 2015. 2015 ESC guidelines for the management of patients with ventricular arrhythmias and the prevention of sudden cardiac death: the task force for the management of patients with ventricular arrhythmias and the prevention of sudden cardiac death of the European society of cardiology (ESC) endorsed by: association for European paediatric and congenital cardiology (AEPC). Eur Heart J. 36(41):2793–2867. 26320108 10.1093/eurheartj/ehv316

[4] Miles C , *et al*. 2023. Subepicardial cardiomyopathy: a disease underlying J-wave syndromes and idiopathic ventricular fibrillation. Circulation. 147(21):1622–1633. 37216437 10.1161/CIRCULATIONAHA.122.061924 PMC11073566

[5] Antzelevitch C , *et al*. 2017. J-wave syndromes expert consensus conference report: emerging concepts and gaps in knowledge. EP Europace. 19(4):665–694. 28431071 10.1093/europace/euw235 PMC5834028

[6] Brugada P, Brugada J. 1992. Right bundle branch block, persistent ST segment elevation and sudden cardiac death: a distinct clinical and electrocardiographic syndrome. J Am Coll Cardiol. 20(6):1391–1396. 1309182 10.1016/0735-1097(92)90253-j

[7] Nademanee K , *et al*. 1997. Arrhythmogenic marker for the sudden unexplained death syndrome in thai men. Circulation. 96(8):2595–2600. 9355899 10.1161/01.cir.96.8.2595

[8] Tan HL, Hofman N, van Langen IM, van der Wal AC, Wilde AAM. 2005. Sudden unexplained death: heritability and diagnostic yield of cardiological and genetic examination in surviving relatives. Circulation. 112(2):207–213. 15998675 10.1161/CIRCULATIONAHA.104.522581

[9] Casado-Arroyo R , *et al*. 2016. Long-term trends in newly diagnosed Brugada syndrome. J Am Coll Cardiol. 68(6):614–623. 27491905 10.1016/j.jacc.2016.05.073

[10] Ben-Haim Y, Asimaki A, Behr ER. 2020. Brugada syndrome and arrhythmogenic cardiomyopathy: overlapping disorders of the connexome? EP Europace. 23(5):653–664. 10.1093/europace/euaa277 33200179

[11] Kapplinger JD , *et al*. 2010. An international compendium of mutations in the SCN5A-encoded cardiac sodium channel in patients referred for Brugada syndrome genetic testing. Heart Rhythm. 7(1):33–46. 20129283 10.1016/j.hrthm.2009.09.069 PMC2822446

[12] Delise P , *et al*. 2018. Cardiac arrest and Brugada syndrome: is drug-induced type 1 ECG pattern always a marker of low risk? Int J Cardiol. 254:142–145. 29180267 10.1016/j.ijcard.2017.10.118

[13] Postema PG . 2018. The value of the sodium channel blocker test in Brugada syndrome and Brugada phenocopy. In: Baranchuk A, editors. Brugada Phenocopy: The Art of Recognizing the Brugada ECG Pattern. Cambridge, MA: Academic Press. p. 21–31.

[14] Ueoka A , *et al*. 2018. Prognostic significance of the sodium channel blocker test in patients with Brugada syndrome. J Am Heart Assoc. 7(10):00. 10.1161/JAHA.118.008617 PMC6015319 29748178

[15] Conte G , *et al*. 2013. Life-threatening ventricular arrhythmias during ajmaline challenge in patients with Brugada syndrome: incidence, clinical features, and prognosis. Heart Rhythm. 10(12):1869–1874. 24055942 10.1016/j.hrthm.2013.09.060

[16] Poli S , *et al*. 2018. Management of untreatable ventricular arrhythmias during pharmacologic challenges with sodium channel blockers for suspected Brugada syndrome. EP Europace. 20(2):234–242. 10.1093/europace/eux092 28521022

[17] Alom MZ , *et al*. 2019. A state-of-the-art survey on deep learning theory and architectures. Electronics. 8(3):292.

[18] Hussain H, Fatt LL. 2007. Efficient ECG signal classification using sparsely connected radial basis function neural network. Proceeding of the 6th WSEAS International Conference on Circuits, Systems, Electronics, Control and Signal Processing. p. 412–416.

[19] Dallali A, Kachouri A, Samet M. 2011. Classification of cardiac arrhythmia using WT, HRV, and fuzzy C-means clustering. Signal Processing: An Int. J.(SPJI). 5(3):101–109.

[20] Homaeinezhad MR , *et al*. 2012. ECG arrhythmia recognition via a neuro-SVM/KNN hybrid classifier with virtual QRS image-based geometrical features. Expert Syst Appl. 39(2):2047–2058.

[21] Zhang Z, Dong J, Luo X, Choi K-S, Wu X. 2014. Heartbeat classification using disease-specific feature selection. Comput Biol Med. 46(1):79–89. 24529208 10.1016/j.compbiomed.2013.11.019

[22] Elhaj FA, Salim N, Harris AR, Swee TT, Ahmed T. 2016. Arrhythmia recognition and classification using combined linear and nonlinear features of ECG signals. Comput Methods Programs Biomed. 127:52–63. 27000289 10.1016/j.cmpb.2015.12.024

[23] Al Rahhal MM , *et al*. 2016. Deep learning approach for active classification of electrocardiogram signals. Inf Sci (Ny). 345:340–354.

[24] Li H, Yuan D, Ma X, Cui D, Cao L. 2017. Genetic algorithm for the optimization of features and neural networks in ECG signals classification. Sci Rep. 7(1):1–12. 28139677 10.1038/srep41011 PMC5282533

[25] Mathews SM, Kambhamettu C, Barner KE. 2018. A novel application of deep learning for single-lead ECG classification. Comput Biol Med. 99(May 2017):53–62. 29886261 10.1016/j.compbiomed.2018.05.013

[26] Mondéjar-Guerra V, Novo J, Rouco J, Penedo MG, Ortega M. 2019. Heartbeat classification fusing temporal and morphological information of ECGs via ensemble of classifiers. Biomed Signal Process Control. 47:41–48.

[27] Attia ZI , *et al*. 2019. Screening for cardiac contractile dysfunction using an artificial intelligence–enabled electrocardiogram. Nat Med. 25(1):70–74. 30617318 10.1038/s41591-018-0240-2

[28] Attia ZI , *et al*. 2019. An artificial intelligence-enabled ECG algorithm for the identification of patients with atrial fibrillation during sinus rhythm: a retrospective analysis of outcome prediction. Lancet. 394(10201):861–867. 31378392 10.1016/S0140-6736(19)31721-0

[29] Grogan M , *et al*. 2021. Artificial intelligence–enhanced electrocardiogram for the early detection of cardiac amyloidosis. Mayo Clin Proc. 96(11):2768–2778. 34218880 10.1016/j.mayocp.2021.04.023

[30] Jentzer JC, Kashou AH, Murphree DH. 2023. Clinical applications of artificial intelligence and machine learning in the modern cardiac intensive care unit. Intell-Based Med. 7:100089.

[31] Mincholé A, Rodriguez B. 2019. Artificial intelligence for the electrocardiogram. Nat Med. 25(1):22–23. 30617324 10.1038/s41591-018-0306-1

[32] Dimitri GM et al 2021. A preliminary evaluation of Echo State Networks for Brugada syndrome classification. In: et al, editors. 2021 IEEE Symposium Series on Computational Intelligence (SSCI). Orlando, FL, USA: IEEE. p. 01–08.

[33] Liu C-M , *et al*. 2022. A deep learning–enabled electrocardiogram model for the identification of a rare inherited arrhythmia: Brugada syndrome. Can J Cardiol. 38(2):152–159. 34461230 10.1016/j.cjca.2021.08.014

[34] Liao S , *et al*. 2022. Use of wearable technology and deep learning to improve the diagnosis of Brugada syndrome. Clin Electrophysiol. 8(8):1010–1020. 10.1016/j.jacep.2022.05.003 35981788

[35] Nakamura T, Aiba T, Shimizu W, Furukawa T, Sasano T. 2022. Prediction of the presence of ventricular fibrillation from a Brugada electrocardiogram using artificial intelligence. Circ J. 87:1007–1014. 36372400 10.1253/circj.CJ-22-0496

[36] Sun X, Xu W. 2014. Fast implementation of Delong’s algorithm for comparing the areas under correlated receiver operating characteristic curves. IEEE Signal Process Lett. 21(11):1389–1393.

[37] Jeske DR, Smith S. 2018. Maximizing the usefulness of statistical classifiers for two populations with illustrative applications. Stat Methods Med Res. 27(8):2344–2358. 27920365 10.1177/0962280216680244

[38] Youden WJ . 1950. Index for rating diagnostic tests. Cancer. 3(1):32–35. 15405679 10.1002/1097-0142(1950)3:1<32::aid-cncr2820030106>3.0.co;2-3

[39] Brugada J, Campuzano O, Arbelo E, Sarquella-Brugada G, Brugada R. 2018. Present status of Brugada syndrome: JACC state-of-the-art review. J Am Coll Cardiol. 72(9):1046–1059. 30139433 10.1016/j.jacc.2018.06.037

[40] Giustetto C, Cerrato N, Gaita F. 2018. Drug-induced type 1 Brugada ECG: lights and shadows. Int J Cardiol. 254:170–171. 29407085 10.1016/j.ijcard.2017.12.044

[41] Bezzina CR , *et al*. 2003. Compound heterozygosity for mutations (w156x and r225w) in scn5a associated with severe cardiac conduction disturbances and degenerative changes in the conduction system. Circ Res. 92(2):159–168. 12574143 10.1161/01.res.0000052672.97759.36

[42] Riera ARP, Antzelevitch C, Schapacknik E, Dubner S, Ferreira C. 2005. Is there an overlap between Brugada syndrome and arrhythmogenic right ventricular cardiomyopathy/dysplasia? J Electrocardiol. 38(3):260–263. 16003713 10.1016/j.jelectrocard.2005.03.009 PMC1479891

[43] Zaklyazminskaya E, Dzemeshkevich S. 2016. The role of mutations in the SCN5A gene in cardiomyopathies. Biochim Biophys Acta, Mol Cell Res. 1863(7):1799–1805. 10.1016/j.bbamcr.2016.02.014 26916278

[44] Mango R , *et al*. 2016. Next generation sequencing and linkage analysis for the molecular diagnosis of a novel overlapping syndrome characterized by hypertrophic cardiomyopathy and typical electrical instability of Brugada syndrome. Circ J. 80(4):938–949. 26960954 10.1253/circj.CJ-15-0685

[45] Chevallier S , *et al*. 2011. New electrocardiographic criteria for discriminating between Brugada types 2 and 3 patterns and incomplete right bundle branch block. J Am Coll Cardiol. 58(22):2290–2298. 22093505 10.1016/j.jacc.2011.08.039

[46] Gottschalk BH , *et al*. 2016. Expert cardiologists cannot distinguish between Brugada phenocopy and Brugada syndrome electrocardiogram patterns. EP Europace. 18(7):1095–1100. 10.1093/europace/euv278 26498159

[47] Ohkubo K , *et al*. 2011. A new criteria differentiating type 2 and 3 Brugada patterns from ordinary incomplete right bundle branch block. Int Heart J. 52(3):159–163. 21646738 10.1536/ihj.52.159

[48] Serra G , *et al*. 2014. New electrocardiographic criteria to differentiate the type-2 Brugada pattern from electrocardiogram of healthy athletes with r’-wave in leads V1/V2. EP Europace. 16(11):1639–1645. 10.1093/europace/euu025 24603955

[49] van der Ree MH . 2021. The β-angle can help guide clinical decisions in the diagnostic work-up of patients suspected of Brugada syndrome: a validation study of the β-angle in determining the outcome of a sodium channel provocation test. EP Europace. 23(12):2020–2028. 10.1093/europace/euab128 PMC8651167 34125232

[50] Okin PM, Anderson KM, Levy D, Kligfield P. 1991. Heart rate adjustment of exercise-induced ST segment depression. Improved risk stratification in the Framingham Offspring study. Circulation. 83(3):866–874. 1999037 10.1161/01.cir.83.3.866

[51] Bhalla V , *et al*. 2005. Diagnostic ability of b-type natriuretic peptide and impedance cardiography: testing to identify left ventricular dysfunction in hypertensive patients. Am J Hypertens. 18(2):73–81. 10.1016/j.amjhyper.2004.11.044 15752936

[52] Chen Y , *et al*. 2016. Pax1 and sox1 methylation as an initial screening method for cervical cancer: a meta-analysis of individual studies in asians. Ann Transl Med. 4(19):365–365. 27826568 10.21037/atm.2016.09.30 PMC5075846

[53] Wu J-T , *et al*. 2017. CHADS2 and CHA2DS2-VASc scores predict the risk of ischemic stroke outcome in patients with interatrial block without atrial fibrillation. J Atheroscler Thromb. 24(2):176–184. 27301462 10.5551/jat.34900 PMC5305678

[54] Ciconte G , *et al*. 2020. Brugada syndrome genetics is associated with phenotype severity. Eur Heart J. 42(11):1082–1090. 10.1093/eurheartj/ehaa942 PMC7955973 33221895

[55] Sieira J , *et al*. 2017. A score model to predict risk of events in patients with Brugada syndrome. Eur Heart J. 38(22):1756–1763. 28379344 10.1093/eurheartj/ehx119

[56] Pappone C , *et al*. 2009. New-onset atrial fibrillation as first clinical manifestation of latent Brugada syndrome: prevalence and clinical significance. Eur Heart J. 30(24):2985–2992. 19696190 10.1093/eurheartj/ehp326

[57] Li W , *et al*. 2018. SCN5A variants: association with cardiac disorders. Front Physiol. 9:1372. 30364184 10.3389/fphys.2018.01372 PMC6191725

[58] Cerrone M, Costa S, Delmar M. 2022. The genetics of Brugada syndrome. Annu Rev Genomics Hum Genet. 23:255–274. 35567276 10.1146/annurev-genom-112921-011200

[59] Conte G , *et al*. 2016. Brugada syndrome and early repolarisation: distinct clinical entities or different phenotypes of the same genetic disease? Arrhythmia Electrophysiol Rev. 5(2):84. 10.15420/AER.2016.23.2 PMC5016597 27617086

[60] Tadros R , *et al*. 2019. Predicting cardiac electrical response to sodium-channel blockade and Brugada syndrome using polygenic risk scores. Eur Heart J. 40(37):3097–3107. 31504448 10.1093/eurheartj/ehz435 PMC6769824

[61] Benjamens S, Dhunnoo P, Meskó B. 2020. The state of artificial intelligence-based FDA-approved medical devices and algorithms: an online database. npj Digital Medicine. 3(1):118. 32984550 10.1038/s41746-020-00324-0 PMC7486909

