# Supplementary material for: Deep learning unmasks the ECG signature of Brugada syndrome
Source: PNAS Nexus. 2023 Oct 13;2(11):pgad327. doi: 10.1093/pnasnexus/pgad327 (PMC10627411; doi:10.1093/pnasnexus/pgad327)
Supplement: pgad327_Supplementary_Data [file pgad327_supplementary_data.pdf]

PNAS nexus

Supplementary Information for

Deep Learning Unmasks the ECG Signature of Brugada Syndrome

Luke Melo[1], Giuseppe Ciconte[2], Ashton Christy[1], Valeria Borrelli[2], Gabriele Vicedomini[2], Luigi Anastasia[3,4], Carlo Pappone[2,3] and Edward Grant[1]

[1]Department of Chemistry, University of British Columbia, Vancouver BC V6T 1Z1, Canada
[2]Arrhythmia and Electrophysiology Center, IRCCS Policlinico San Donato, San Donato Milanese 20097, Milano, Italy
[3]Vita-Salute San Raffaele University, Milano, 20132, Italy
[4]Laboratory of Stem Cells for Tissue Engineering, IRCCS Policlinico San Donato, Milano, 20132, Italy

Corresponding authors: Luigi Anastasia, Carlo Pappone and Edward Grant
Email: anastasia.luigi@hsr.it, carlo.pappone@af-ablation.org, edgrant@chem.ubc.ca

Supplementary Information list of contents:

Supplemental Methods -

- The ECG Processing Pipeline: A Journey from ECG to Diagnosis
    - Context
    - Conventional CNN/RNN Approach
    - R-Peak Based ECG Segmentation using Wavelet Transform
    - Autoencoder for Fast R-Peak Detection
        - Figure S1
    - Autoencoder Details
        - Figure S2
    - Autoencoder Output Post-Processing
- Detailed Methods
    - Filtering and Denoising
    - Detection of Heartbeats
    - Outlier Identification
    - Neural Networks
    - Calibration and Validation
    - Validation of the 9-Lead DNN Model
- Bandpass Filtering of Electrical Noise
        - Figure S3
- Statistical Treament and Superposition of Heartbeats to form Representative Average ECG Waveforms
        - Figures S4, S5
- Detailed Outcomes in the Tests for Overfitting

    - Figure S6
- Confidence Intervals Associated with Statistical Predictions

Supplemental Results -

- Representative ECG Data
    - Figures S7–S13
- Leave One Out Cross Validation (LOOCV) and Ensemble Learning
    - Figure S14
- DNN Feature Importance in the 12 Lead ECG
    - Figure S15
- Details in the Prediction of Brugada Syndrome for the *Claris* Training Cohort
    - Table S1
- Details in the Prediction of Brugada Syndrome for the *Mortara* Validation Cohort
    - Table S2
- Demographics of the Training and Validation Cohorts
    - Tables S3, S4
- Details of the Classification of Brugada Syndrome
    - Table S5
- Consideration of Additional Factors of Variation
    - Tables S6, S7
- Convolutional Neural Network (CNN) Processing
    - Table S8
- Convolutional Neural Network Validation Results
    - Table S9
- Detailed Clinician Results
    - Tables S10, S11
- DNN Performance Comparison with Other Popular Classifiers
    - Table S12

References

The ECG Processing Pipeline: A Journey from ECG to Diagnosis

Context

As its ultimate goal, our automated digital ECG analysis seeks to classify a BrS disease state on the basis of features extracted from a standard ECG trace. Broadly speaking, all algorithms approach this problem in the following way:

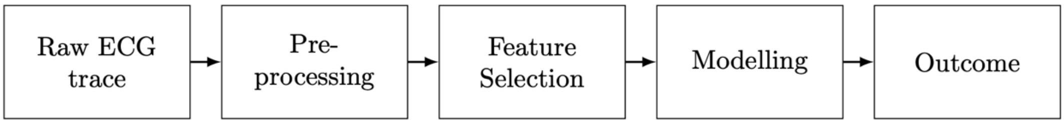

Conventional CNN/RNN Approach

The most common data processing pipeline for ECG analysis applies a convolutional or recurrent neural network (CNN or RNN) directly to the raw ECG trace. This provides the most hands-off approach, because the preprocessing, feature selection and modelling are all streamlined into a single step. Here, the CNN/RNN algorithm generates and optimizes a set of features that it finds best correlated to the outcome. It then feeds this set of features to a fully-connected deep neural network (DNN), which determines the optimal combination and weighting of features to predict the outcome. This strategy comes with its own advantages and disadvantages:

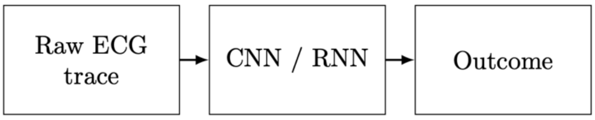

Advantages	Disadvantages
• Simple to implement<br>• Rapid deployment (applicable to streaming data)<br>• Minimal or no data preprocessing needed<br>• Generally yields satisfactory results	• Prolonged model training time<br>• Classifies based on a set of arbitrarily learned features<br>• Black box learning process

R-Peak Based ECG Segmentation using Wavelet Transform

We employ a robust ECG preprocessing strategy that derives a single, high-fidelity representative heartbeat from the full trace for each lead of the input ECG. Our algorithm subsequently classifies upon this basis. A representative heartbeat in our case is the median of a single, complete cardiac cycle, centered near the R peak, as recorded by each lead. We meticulously locate each R peak in an ECG trace by a multi-resolution continuous wavelet transform (CWT) deconstruction. We find this yields the most accurate results, because our CWT procedure identifies parts of the ECG signal in time that exhibit the spatial frequency (sharpness) of the R peak. This function of signal morphology and time requires a significant amount of computational time to generate. But, once it identifies the R peaks located along a trace, the algorithm rapidly segments and stacks each detected beat within a canonical 750 ms window to form a complete captured cardiac cycle. We next perform clustering analysis to remove statistical outliers. Averaging this stack of ECGs produces a very high SNR representative heartbeat. We feed this representative heart beat to a fully connected DNN for classification.

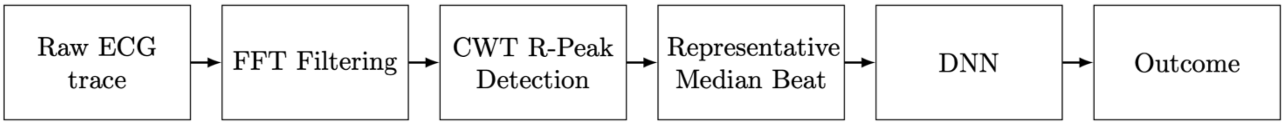

Advantages	Disadvantages
• The high SNR representative heartbeat *is* the feature. It is not learned, but extracted as a persistent property of the raw ECG trace.<br>• Statistically superior performance in classification over CNN / RNN approaches<br>• Quick to train DNN	• Accurate determination of R-peak positions requires elaborate data processing. This step forms the computational bottleneck.<br>• Cannot be applied to streaming data (no real-time analysis).<br>• The code is complex.

Autoencoder for Fast R-Peak Detection

Owing to the computational expense of CWT calculations, we developed a neural network-based approach to identifying and localizing R peaks. Here, we sought to combine the accuracy afforded by the combined representative beat and DNN approach with the computational efficiency of the CNN approach. Unfortunately, a CNN by itself struggles to learn a diagnostically useful set of features to form the representative beat. To solve this problem, we accelerated the process of R peak detection while maintaining the accuracy of the CWT algorithm.

An autoencoder (1) is a convolutional neural network-based learning hierarchy that learns a path leading directly from a raw ECG trace to a segmentation map of R peak locations. The segmentation map in our case forms a classification data structure of the same dimensionality as an ECG trace that represents R peaks locations as 1 and every other point as 0. We apply the AE in place of the CWT algorithm to locate R peaks.

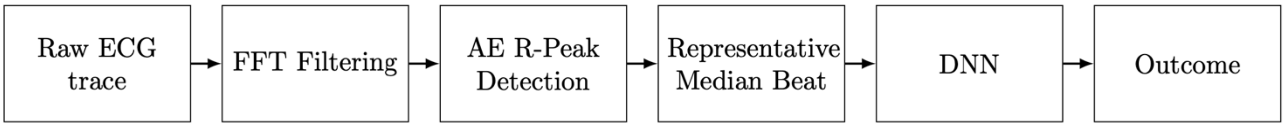

Advantages	Disadvantages
• Significant improvement in deployment time<br>• Minimal to no ECG data preprocessing needed<br>• Produces representative beat consistent with CWT method	• Requires some postprocessing to converge on unique R peak timestamps<br>• False-positive identification of T waves can occur

Across 1,865 ECGs, the R peak location computation time with CWT for an 8 second trace is on average 20.3 seconds, which is about 2.5 times longer than ECG trace itself. With the same set of ECGs, the computation time for the autoencoder method is on average 0.011 seconds, which is 727 times shorter than duration of the ECG trace.

This enormous speedup in processing time opens the door to perform real-time monitoring of heartbeats with very low computational expense. Any currently available ECG machine has the onboard computational power to apply our algorithm in real time. Some applications could include:

- real-time monitoring of ST elevation in the operating room
- real-time processing on wearable devices
- lightweight cloud computing for uploaded ECGs (can process an enormous bandwidth of ECGs, probably a few hundred per second on a regular desktop computer)

Beyond the diagnosis of BrS, this enables the ability to create representative heart beats for enormous datasets of millions of subjects in a reasonable time frame. We have shown that the representative heart beat is a superior machine learning input feature than a CNN / RNN can find for itself.

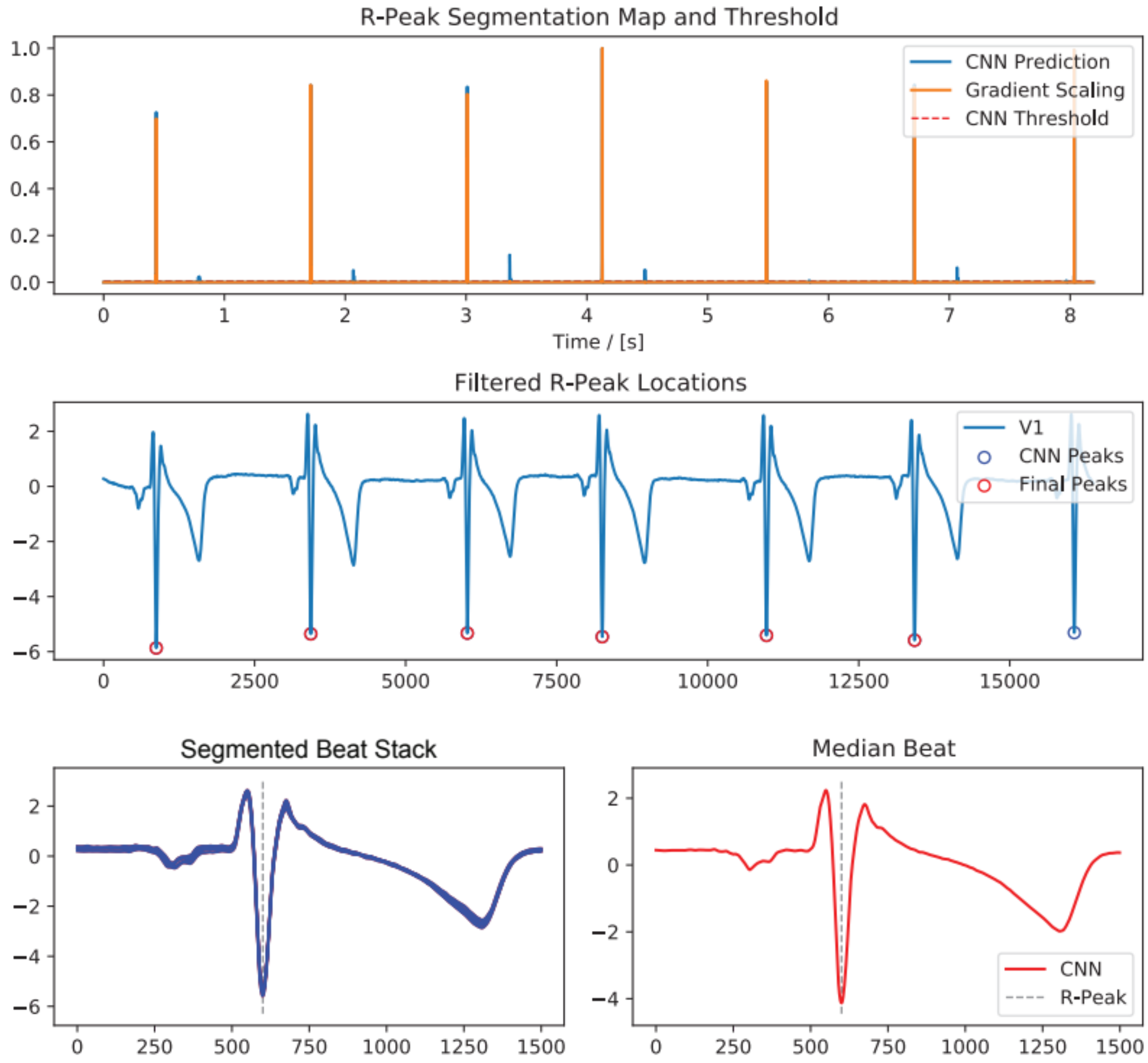

Figure S1: Example of R peak location results using the autoencoder algorithm. (2) Segmentation map, where identified R peaks are assigned a value of 1, and all other points 0. (Middle) Raw ECG trace with R peak locations identified. (Bottom left) Stack of segmented heartbeats, based on R peak locations. (Bottom right) The same stack of segmented heartbeats, after cluster analysis has identified and removed outliers.

Autoencoder Details

The autoencoder implemented closely follows the algorithm presented by Zahid *et. al.* 2021[1].

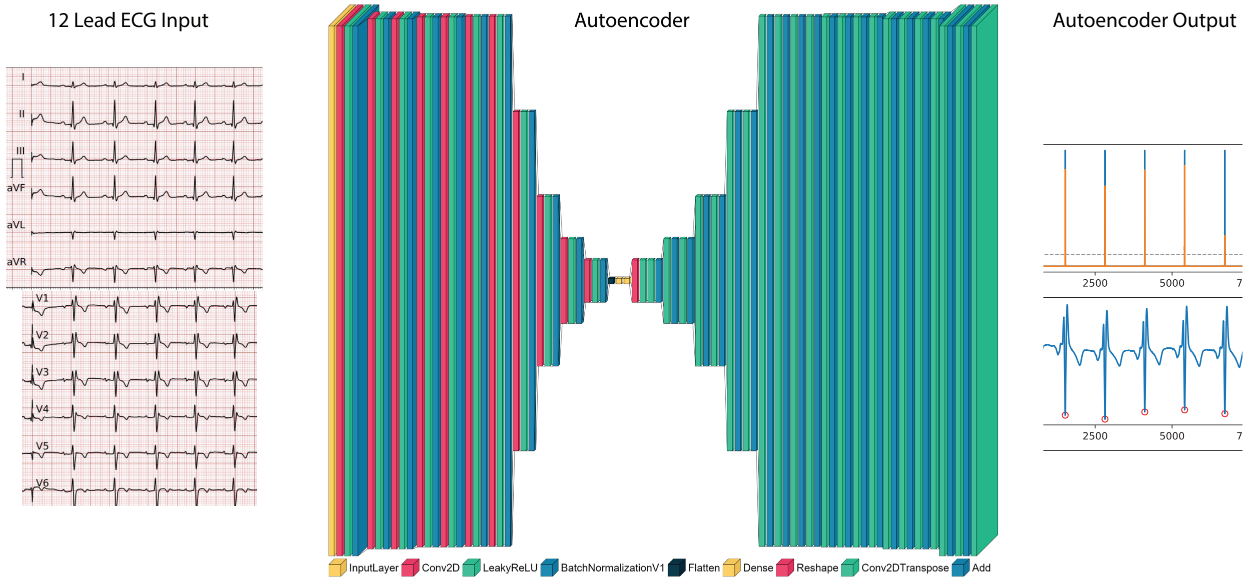

Layer (type)	Output Shape	Param #	Connected to
input_1 (InputLayer)	(None, 12, 16384, 1)	0	
conv2d_1 (Conv2D)	(None, 12, 8192, 9)	27	input_1[0][0]
leaky_re_lu_1 (LeakyReLU)	(None, 12, 8192, 9)	0	conv2d_1[0][0]
batch_normalization_1 (BatchNor	(None, 12, 8192, 9)	36	leaky_re_lu_1[0][0]
conv2d_2 (Conv2D)	(None, 4, 4096, 9)	495	batch_normalization_1[0][0]
leaky_re_lu_2 (LeakyReLU)	(None, 4, 4096, 9)	0	conv2d_2[0][0]
batch_normalization_2 (BatchNor	(None, 4, 4096, 9)	36	leaky_re_lu_2[0][0]
conv2d_3 (Conv2D)	(None, 4, 2048, 6)	114	batch_normalization_2[0][0]
leaky_re_lu_3 (LeakyReLU)	(None, 4, 2048, 6)	0	conv2d_3[0][0]
batch_normalization_3 (BatchNor	(None, 4, 2048, 6)	24	leaky_re_lu_3[0][0]
conv2d_4 (Conv2D)	(None, 2, 1024, 6)	150	batch_normalization_3[0][0]
leaky_re_lu_4 (LeakyReLU)	(None, 2, 1024, 6)	0	conv2d_4[0][0]
batch_normalization_4 (BatchNor	(None, 2, 1024, 6)	24	leaky_re_lu_4[0][0]
conv2d_5 (Conv2D)	(None, 2, 512, 3)	39	batch_normalization_4[0][0]
leaky_re_lu_5 (LeakyReLU)	(None, 2, 512, 3)	0	conv2d_5[0][0]
batch_normalization_5 (BatchNor	(None, 2, 512, 3)	12	leaky_re_lu_5[0][0]
conv2d_6 (Conv2D)	(None, 1, 256, 3)	39	batch_normalization_5[0][0]
leaky_re_lu_6 (LeakyReLU)	(None, 1, 256, 3)	0	conv2d_6[0][0]
batch_normalization_6 (BatchNor	(None, 1, 256, 3)	12	leaky_re_lu_6[0][0]
conv2d_7 (Conv2D)	(None, 1, 128, 3)	21	batch_normalization_6[0][0]
leaky_re_lu_7 (LeakyReLU)	(None, 1, 128, 3)	0	conv2d_7[0][0]
batch_normalization_7 (BatchNor	(None, 1, 128, 3)	12	leaky_re_lu_7[0][0]
conv2d_8 (Conv2D)	(None, 1, 64, 3)	21	batch_normalization_7[0][0]
leaky_re_lu_8 (LeakyReLU)	(None, 1, 64, 3)	0	conv2d_8[0][0]
batch_normalization_8 (BatchNor	(None, 1, 64, 3)	12	leaky_re_lu_8[0][0]
conv2d_9 (Conv2D)	(None, 1, 32, 3)	21	batch_normalization_8[0][0]
leaky_re_lu_9 (LeakyReLU)	(None, 1, 32, 3)	0	conv2d_9[0][0]
batch_normalization_9 (BatchNor	(None, 1, 32, 3)	12	leaky_re_lu_9[0][0]
conv2d_10 (Conv2D)	(None, 1, 16, 3)	21	batch_normalization_9[0][0]
leaky_re_lu_10 (LeakyReLU)	(None, 1, 16, 3)	0	conv2d_10[0][0]
batch_normalization_10 (BatchNo	(None, 1, 16, 3)	12	leaky_re_lu_10[0][0]
conv2d_11 (Conv2D)	(None, 1, 8, 3)	21	batch_normalization_10[0][0]
leaky_re_lu_11 (LeakyReLU)	(None, 1, 8, 3)	0	conv2d_11[0][0]
batch_normalization_11 (BatchNo	(None, 1, 8, 3)	12	leaky_re_lu_11[0][0]
flatten_1 (Flatten)	(None, 24)	0	batch_normalization_11[0][0]
dense_1 (Dense)	(None, 24)	600	flatten_1[0][0]
dense_2 (Dense)	(None, 24)	600	dense_1[0][0]
reshape_1 (Reshape)	(None, 1, 8, 3)	0	dense_2[0][0]
conv2d_transpose_1 (Conv2DTrans	(None, 1, 8, 3)	21	reshape_1[0][0]
leaky_re_lu_12 (LeakyReLU)	(None, 1, 8, 3)	0	conv2d_transpose_1[0][0]
batch_normalization_12 (BatchNo	(None, 1, 8, 3)	12	leaky_re_lu_12[0][0]
conv2d_transpose_2 (Conv2DTrans	(None, 1, 16, 3)	21	batch_normalization_12[0][0]
add_1 (Add)	(None, 1, 16, 3)	0	conv2d_10[0][0]<br>conv2d_transpose_2[0][0]
leaky_re_lu_13 (LeakyReLU)	(None, 1, 16, 3)	0	add_1[0][0]
batch_normalization_13 (BatchNo	(None, 1, 16, 3)	12	leaky_re_lu_13[0][0]
conv2d_transpose_3 (Conv2DTrans	(None, 1, 32, 3)	21	batch_normalization_13[0][0]
add_2 (Add)	(None, 1, 32, 3)	0	conv2d_9[0][0]<br>conv2d_transpose_3[0][0]
leaky_re_lu_14 (LeakyReLU)	(None, 1, 32, 3)	0	add_2[0][0]
batch_normalization_14 (BatchNo	(None, 1, 32, 3)	12	leaky_re_lu_14[0][0]
conv2d_transpose_4 (Conv2DTrans	(None, 1, 64, 3)	21	batch_normalization_14[0][0]
add_3 (Add)	(None, 1, 64, 3)	0	conv2d_8[0][0]<br>conv2d_transpose_4[0][0]
leaky_re_lu_15 (LeakyReLU)	(None, 1, 64, 3)	0	add_3[0][0]
batch_normalization_15 (BatchNo	(None, 1, 64, 3)	12	leaky_re_lu_15[0][0]
conv2d_transpose_5 (Conv2DTrans	(None, 1, 128, 3)	21	batch_normalization_15[0][0]
add_4 (Add)	(None, 1, 128, 3)	0	conv2d_7[0][0]<br>conv2d_transpose_5[0][0]
leaky_re_lu_16 (LeakyReLU)	(None, 1, 128, 3)	0	add_4[0][0]
batch_normalization_16 (BatchNo	(None, 1, 128, 3)	12	leaky_re_lu_16[0][0]
conv2d_transpose_6 (Conv2DTrans	(None, 1, 256, 3)	21	batch_normalization_16[0][0]
add_5 (Add)	(None, 1, 256, 3)	0	conv2d_6[0][0]<br>conv2d_transpose_6[0][0]
leaky_re_lu_17 (LeakyReLU)	(None, 1, 256, 3)	0	add_5[0][0]
batch_normalization_17 (BatchNo	(None, 1, 256, 3)	12	leaky_re_lu_17[0][0]
conv2d_transpose_7 (Conv2DTrans	(None, 2, 512, 3)	39	batch_normalization_17[0][0]
add_6 (Add)	(None, 2, 512, 3)	0	conv2d_5[0][0]<br>conv2d_transpose_7[0][0]
leaky_re_lu_18 (LeakyReLU)	(None, 2, 512, 3)	0	add_6[0][0]
batch_normalization_18 (BatchNo	(None, 2, 512, 3)	12	leaky_re_lu_18[0][0]
conv2d_transpose_8 (Conv2DTrans	(None, 2, 1024, 6)	42	batch_normalization_18[0][0]
add_7 (Add)	(None, 2, 1024, 6)	0	conv2d_4[0][0]<br>conv2d_transpose_8[0][0]
leaky_re_lu_19 (LeakyReLU)	(None, 2, 1024, 6)	0	add_7[0][0]
batch_normalization_19 (BatchNo	(None, 2, 1024, 6)	24	leaky_re_lu_19[0][0]
conv2d_transpose_9 (Conv2DTrans	(None, 4, 2048, 6)	150	batch_normalization_19[0][0]
add_8 (Add)	(None, 4, 2048, 6)	0	conv2d_3[0][0]<br>conv2d_transpose_9[0][0]
leaky_re_lu_20 (LeakyReLU)	(None, 4, 2048, 6)	0	add_8[0][0]
batch_normalization_20 (BatchNo	(None, 4, 2048, 6)	24	leaky_re_lu_20[0][0]
conv2d_transpose_10 (Conv2DTran	(None, 4, 4096, 9)	117	batch_normalization_20[0][0]
add_9 (Add)	(None, 4, 4096, 9)	0	conv2d_2[0][0]<br>conv2d_transpose_10[0][0]
leaky_re_lu_21 (LeakyReLU)	(None, 4, 4096, 9)	0	add_9[0][0]
batch_normalization_21 (BatchNo	(None, 4, 4096, 9)	36	leaky_re_lu_21[0][0]
conv2d_transpose_11 (Conv2DTran	(None, 12, 8192, 9)	495	batch_normalization_21[0][0]
add_10 (Add)	(None, 12, 8192, 9)	0	conv2d_1[0][0]<br>conv2d_transpose_11[0][0]
leaky_re_lu_22 (LeakyReLU)	(None, 12, 8192, 9)	0	add_10[0][0]
batch_normalization_22 (BatchNo	(None, 12, 8192, 9)	36	leaky_re_lu_22[0][0]
conv2d_transpose_12 (Conv2DTran	(None, 12, 16384, 1)	19	batch_normalization_22[0][0]

Total params: 3,565
Trainable params: 3,361
Non-trainable params: 204

Figure S2: Autoencoder algorithm description. (2) Graphical representation of the data pipeline, from raw ECG traces (left) to R peak segmentation map (right). (Bottom) Detailed description of AE CNN structure.

Autoencoder Output Post-Processing

The following steps outline the post-processing strategy for filtering the output of the autoencoder to determine a discrete set of R peak locations.

1. Apply a threshold to the CNN results to select peaks above the baseline. A standard deviation threshold of $\sigma = 0.5$ is used.
2. Apply a low-pass filter with a cutoff frequency of 30 Hz to the second derivative of the ECG signal using a Butterworth filter of order 9.
3. Multiply the filtered second derivative by the original CNN prediction and normalize the result. This step helps reduce the identification of false positive P and T waves.
4. Choose the V1 lead from the ECG data.
5. Remove R peaks that are within 20 ms of the beginning or end of the signal, as these often are incomplete heartbeats.
6. Use basin-hopping to find unique R peak locations.
   - For each R peak location, find the local minimum within a window centered around the R peak.
   - Update the R peak locations based on the local minima found.
   - Remove duplicates and ensure that the R peak locations are within the valid range.
   - Basin-hopping is performed for 10 iterations.
7. Apply a 400 ms exclusivity rule to remove duplicates and nearby false positives.
8. Calculate the heart rate (HR) and heart rate variability (HRV) from the final R-peak locations.
9. Create a representative beat for each subject by chopping and stacking ECG signals around the R peaks.
   - For each R peak location, extract a window of the ECG signal centered near the R-peak. Typically this window is 750 ms. The window is centered slightly after the R peak location, due to the typically longer duration of the QT interval compared to the PR interval.
   - Store the extracted windows in repstack_12lead.
10. Apply cluster analysis to remove outlier beats, then calculate the representative beat taking the median of the remainder.
11. If clustering_opt is set to False, calculate the representative beat by taking the median across all beats.

Detailed Methods

Filtering and Denoising

We first apply a sequence of infinite impulse response (IIR) filters to each ECG trace, treating them as electronic signals. To this purpose, we apply a fast Fourier transform (FFT) to the traces and use a peak-finding algorithm on the resulting power spectrum to identify anomalous frequencies. We suppress these with a series of bandstop filters with widths of 3 Hz. We then apply another series of bandstop filters to remove 50 Hz AC hum (2) and its overtones from the ECG. Finally, we apply a highpass filter, centered at 0.5 Hz, to remove baseline drift from the trace (1); this varies according to the selected onboard filters and the electromagnetic interference (EMI) environment of the ECG instrument. The result is a denoised ECG signal with a flat baseline at 0 mV. Figure S3 provides an example of the power spectrum of an ECG trace before and after denoising.

Detection of Heartbeats

In order to generate consistent representative heartbeats, we define a canonical window of fixed length that fully encompasses the typical cardiac cycle for every subject. For the purposes of this study, we set this window to 750 ms, corresponding to a heart rate of 80 BPM, typical of our cohort. We choose the R peak as the reference point of our canonical window owing to its consistently sharp morphology.

To identify and localize R peaks, we apply a CNN based autoencoder described previously. For a given lead the set of R peak positions serve to align the heartbeat waveforms to form an average.

Having established the R peak positions, we segment each trace to fit a canonical 750 ms window, individually defined around each R peak. As such, these canonical windows capture each detected heartbeat regardless of heart rate, though some overlapping information may be captured in subjects with elevated heart rates. We align the R peaks to 300 ms from window onset, a slight offset from the window's midpoint, in order to fully capture the systole. This offset also helps to account for subjects with longer QT intervals, whose complete cardiac cycles might not otherwise be captured within our canonical window. The result is a two-dimensional stack of individual heartbeats for each subject, as shown in Figure 3a. Here, the R peak positions also provide the information necessary to synchronize the positions of traces in the measurement window before fusion.

Outlier Identification

The two-dimensional stack of ECG traces for each lead may include statistical outliers for several reasons, such as patient movement, irregularities in a single cardiac cycle (e.g., premature beat), change in shape of the ECG that causes the detection algorithm to misidentify the R peak, or a change in the trace baseline that has escaped the FFT highpass filter. Figure S4 shows some examples. These degrade the fidelity of the representative beat. Removing these outliers improves the suitability of the dataset for calibration purposes.

To identify statistical outliers in each ECG stack, we apply a series of outlier detection algorithms and reject individual heartbeats identified as such. We first apply principal component analysis (PCA), which projects the data onto an orthogonal basis, in which each dimension (principal component) represents a unique contribution to the overall variance in the data. Using Hotelling's T^2 statistic (3), we define a 95% confidence region in the first two principal component spaces (see Figure S4A. The critical distance of the confidence region is defined as follows:

$$T = \sqrt{\sigma^2(s) * \left[\frac{A(N^2-1)}{N(N-A)}\right] * F_{\text{crit},\alpha=0.05}(N-A,A)} \quad (1)$$

where s is the principal component score matrix of the two-dimensional stack of ECG segments, N is the number of individual heartbeats in the ECG stack (i.e., population size), A is the number of principal components in the model, and F_{crit} is the critical value of the F–distribution with the given degrees of freedom at 95% confidence. Heartbeats in the ECG stack that lie outside this confidence region in the first two principal components are marked as outliers, and are rejected from the dataset. These outliers are transient irregularities in the ECG trace, which can be caused by myriad factors, including patient movement or momentary electrical artifacts. In cases where subjects present with high intra-beat variability, the confidence region would be correspondingly larger, encompassing more of that variance.

We next calculate the leverage h that each heartbeat i in the ECG stack has on the principal component model as follows:

$$h_i = \frac{1}{N} + \sum_a \frac{s_{i,a}^2}{s_a^{\mathsf{T}} \cdot s_a} \tag{2}$$

where a is the principal component ($a \in [1, A]$), s is the principal component score matrix of the ECG stack, and N is the number of individual heartbeats in the ECG stack (i.e., population size). Heartbeats whose leverages exceed $\mu + 2\sigma$ are marked as outliers, and are rejected from the dataset.

We then perform a density-based spatial clustering of applications with noise (DBSCAN) (4) analysis on the principal component scores. This analysis uses a k–nearest neighbor algorithm as a distance measure to identify clusters of heartbeats in the ECG stack; this is useful in cases where noisy ECG signals have caused the misidentification of some R-peaks. Heartbeats falling outside the largest identifiable cluster are marked as outliers and may be rejected from the dataset. The resulting corrected segmented traces are shown in Figure 3a. Figure S5 shows an example DBSCAN analysis.

Once outliers have been removed from the ECG stack, we calculate the median value for each stack; this generates a representative heartbeat for each of the twelve ECG leads (Figure 3a). Data from each step outlined above were inserted back into the SQL database for each subject.

Neural Networks

Among a total of 1,455 unique subjects, the DNN optimization procedure accepts 1,154 ECG records (596 Brugada and 558 Control, respectively). We have formed the primary input for developing various DNN models from chosen configurations of X_{ECG} constructed using various combinations of $n_{\mathrm{leads}} \in [1, 12]$ leads. We have down-sampled ECGs in every case from 2000 Hz to 200 Hz, which exceeds the Nyquist limit of 150 Hz. This is consistent with practices reported elsewhere. With a heartbeat sampling window of 750 ms, this sampling interval reduces the dimensionality of the classification problem to n_{leads} *1500/(2000 Hz/200 Hz) = 150*n_{leads}. We have also tested the utility of other factors of variation (FoV) such as age, sex, personal histories of syncope and cardiac arrest, and family histories of sudden death and BrS diagnosis, formatted similarly to the target vector y, and concatenated to the representative fused ECG for each subject.

The neural network consists of a sequence of input, hidden, and output layers, repeated three times, as shown in Figure 3b. The input layer accepts $150 * n_{\mathrm{leads}} + n_{\mathrm{FoV}}$ elements for each subject. Each hidden layer unit incorporates a fully connected layer linking 5 nodes with an L1 kernel regularizer (5), a Gaussian noise regularization layer (6) with a standard deviation of $\sigma = 0.1$, a rectified linear activation unit (ReLU) function (7), and a batch normalization layer (8). The multilayer perceptron formed after three successive applications of this hidden layer, passes to a fully connected classification layer with a sigmoid activation function, in which an adagrad optimizer minimizes the binary cross-entropy function (9). An early stopping algorithm terminate if a local minimum is reached in the output of the binary cross-entropy loss function of the validation data. The algorithm allows for a maximum of 10,000 epochs with a patience of 50 epochs. An isotonic regression function (10) calibrates the predicted probabilities to the distribution of DNN scores (11).

We have performed an extensive grid search optimizing the accuracy of calibration as a function of the structure of the DNN model with respect to the following degrees of freedom. We additionally explored the use of convolutional layers which yielded lower training accuracies than the DNNs trained with representative heart beats. The additional computation time needed to train convolutional neural networks (CNNs) made analysis by LOOCV unattainable for this dataset.

- Nodes: 1, 2, 3, ..., 9, 10
- Layers: 1, 2, 3, ..., 9, 10

- Batch size: 1, 2, 4, ..., 128, 256
- Gradient Descent Optimizers: adam,(12) adagrad,(9) rmsprop, sgd
- Gaussian Noise
- Dropout Layers(13)
- Activation / Batch Normalization Sequence
- Convolutional layers before dense layers

Calibration and Validation

To exercise maximum independence in calibration and validation, we create a separate, distinct neural network modeling problem for every subject. Setting aside one subject in each case as an independent holdout, we partition the remaining ECG database into training and validation subsets using stratified *k*–fold cross-validation with 7 folds (86%: 14% training and validation split). We iteratively optimize weight and bias matrices through backpropagation, using the training subset under supervision of a validation subset. Upon maximizing the binary cross-entropy function, we test the trained DNN with the independent holdout subject using leave-one-out cross-validation (LOOCV), as shown in Figures 3c and S13. We have also explored bootstrapping, though this exhibits little difference generalization when compared to *k*–fold cross-validation.

In order to minimize sampling bias in the training/validation subset, we have carried out 1,154 distinct iterations of the above procedure, in every case tuned by 7–fold cross-validation. This amounts to 8,078 separately calibrated DNNs for each set of lead combinations considered.

Validation of the 9-Lead DNN Model

The text describes a DNN model for the classification of the response to an ajmaline challenge, performed to diagnose BrS; its performance has been confirmed by nearly 2,500 independent validations. Because the local uncontrolled variance of any single ECG measurement vastly exceeds the variation in response between individual ECG instruments, we can expect our model to show comparable performance in the classification of a subsequent consecutive cohort. As a preliminary test of this hypothesis, we collected the electrocardiograms of a new cohort of 405 consecutive subjects admitted to IRCCS Policlinico San Donato for BrS screening. In addition to the standard protocol, including a sodium channel blocker challenge, these subjects underwent ECG examination using Mortara ELI™ 350 ECG machines, recorded using the same high precordial lead placement. We processed the digitized Mortara ECGs according to the previously described procedure, generating downsampled representative heartbeats, and termed this the *Mortara* cohort. We analyzed these 9-lead representative traces to predict sodium channel blocker response using our DNN model, which was trained solely with the training dataset, obtained by the Claris™ system, as previously described in the text.

Bandpass Filtering of Electrical Noise

Figure S3 shows the power spectra of raw and denoised power spectra of an ECG trace as calculated by FFT. Note that the regular, sharp peaks in the raw spectrum (*A*), representing AC hum, are suppressed.

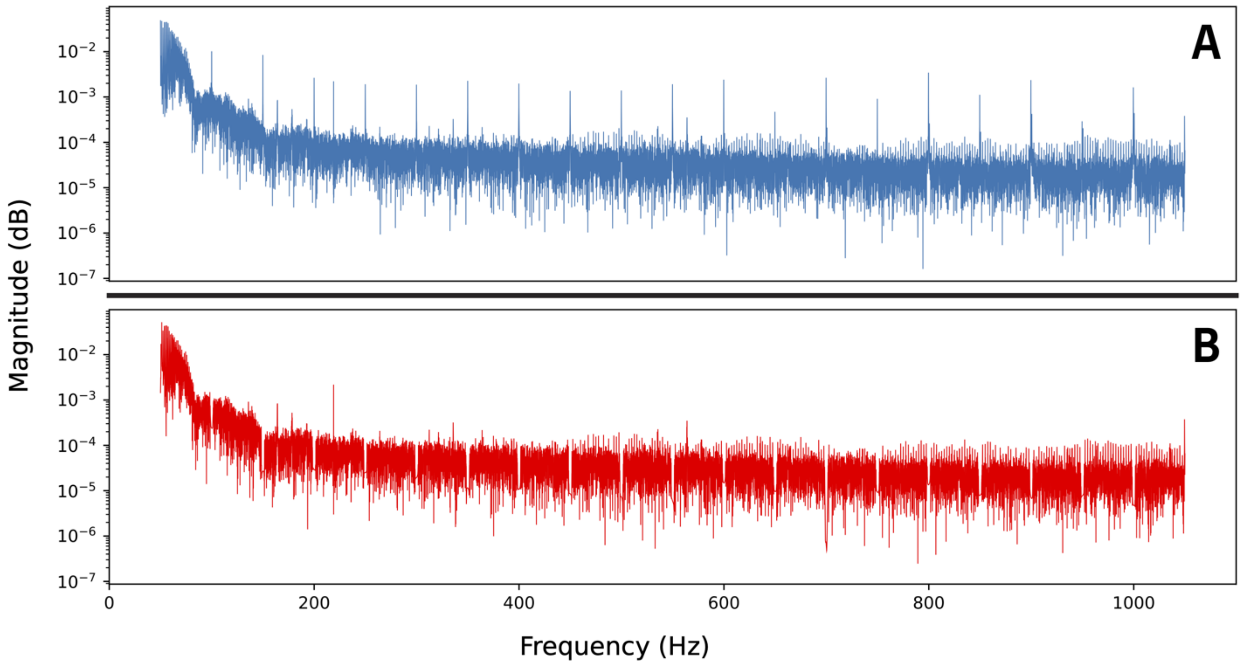

Figure S3. Power spectra of an ECG trace pre- (*A*) and post-denoising (*B*).

Statistical Treatment and Superposition of Heartbeats to Form Representative Average ECG Waveforms

Figures S4A and B show an example of principal component analysis applied to the process of detecting outliers in forming a representative average ECG signal for each lead. Here, blue dots represent accepted traces, while red dots refer to rejected traces, as determined by the process outlined in the main text.

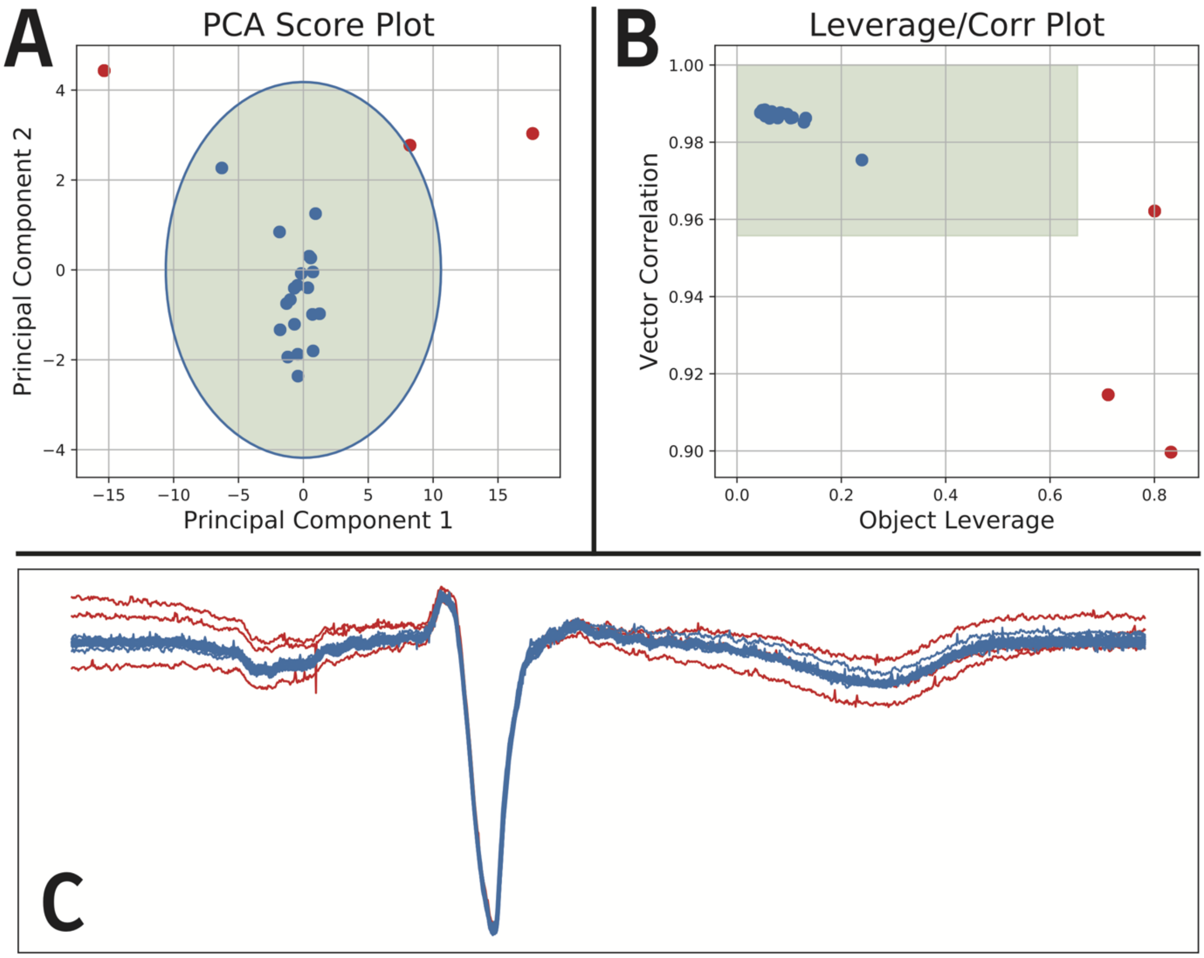

Figure S4. Typical outlier detection plots for a stack of ECG traces. Outliers are indicated in red. *A:* Hotelling's T2 confidence ellipse overlaid on principal components 1 and 2. *B* Leverage *h* plotted against Pearson's correlation coefficient for each ECG segment. *C:* The ECG stack, with rejected traces indicated in red.

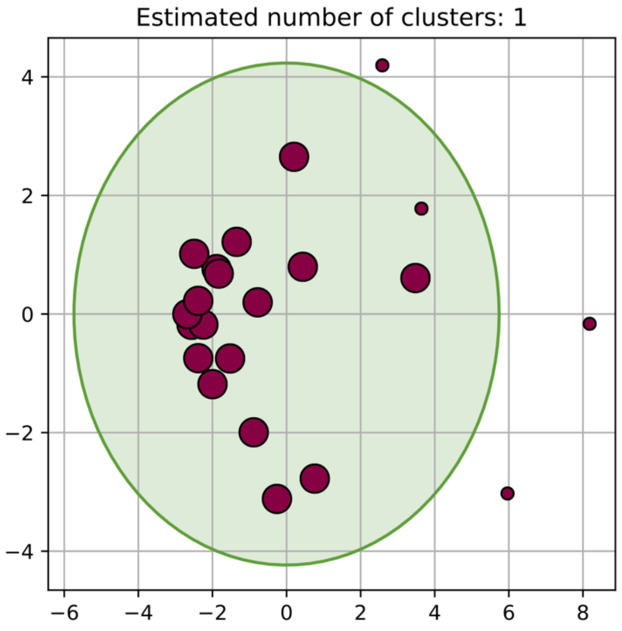

Figure S5. Typical example of a DBSCAN analysis on a stack of ECG traces in PCA space.

Detailed Outcomes in the Tests for Overfitting

The reliable extension of a multivariate classification model to the classification of a new, independent set of holdout subjects requires rigorous tests of overfitting. Figure S6 shows results that certify the absence of overfitting in the development of DNN models classifying ECGs.

Though limited in size from an informatics perspective, these datasets represent the largest available collection of electrocardiograms determinately classified to recognize Brugada Syndrome even in the case of an apparently normal ECG. Our constraints on database size present particular concerns with regard to data handling, and avoiding the definition of networks solely on the basis of self-consistency. Though we employ rigorous cross-validation steps to prevent overfitting, we have also taken three steps to assess if this problem is nonetheless present. As further tests for overfitting, 1) We check for convergence between the training and testing loss functions in the DNN training step. 2) We employ LOOCV. For each subject, we exclude this one subject from the subset of ECG data in the DNN training and validation. Through a 7–fold CV partitioning scheme of the DNN training subset, we apply these DNNs to each reserved subject to generate predictions of BrS diagnosis, along with concomitant DNN scores. 3) As a final test, we train neural networks after having randomly permuted the diagnosis vector. This process creates a model with no deterministic correlation between the ECG data and BrS type 1 pattern; such a model should have no better than a random chance of classifying an ECG correctly (≈50% accuracy). We perform this randomization process dozens of times and have observed that the resulting trained neural networks consistently have accuracies near 50%. Additionally, the DNN model accurately classifies independent ECGs recorded in separate circumstances using the Mortara ELI™ 350 system.

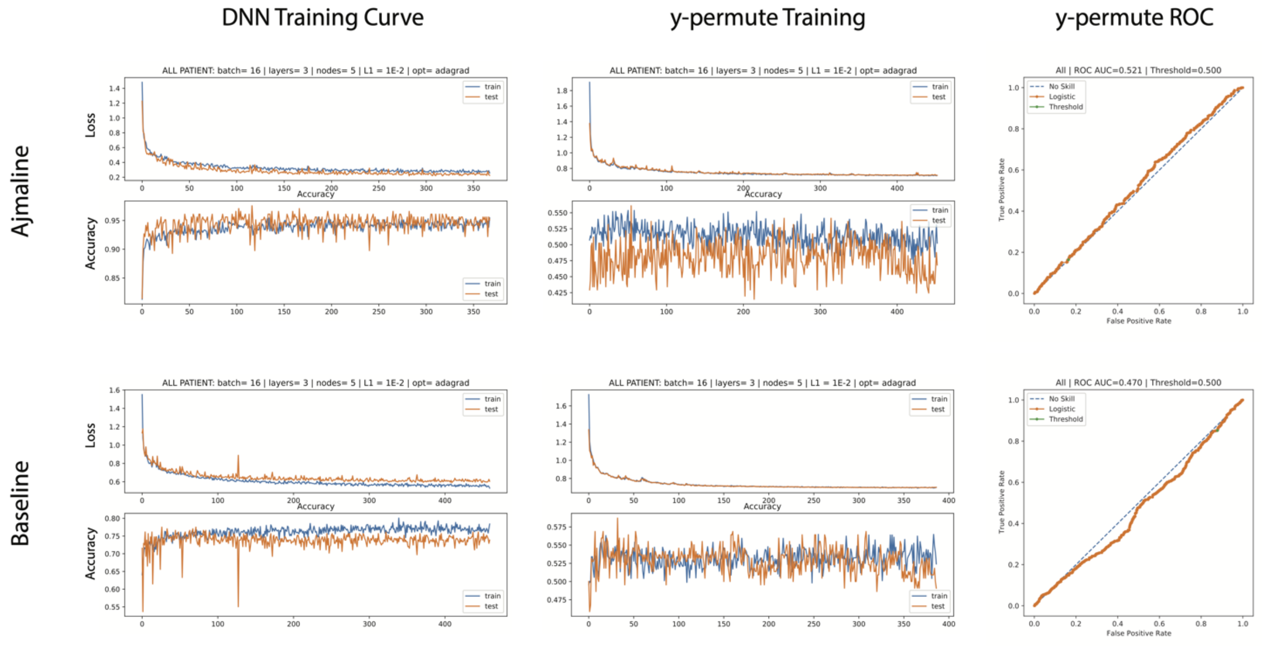

Figure S6. DNN training curves, y-vector permutation overfitting test training curves, and y-vector permutation overfitting test ROCs. For each training curve and dataset, the results of cross-entropy loss function (2) and accuracy (bottom) are shown as a function of training epoch. The resulting LOOCV ROC curve is shown for each dataset under the y-vector permutation overfitting test.

Figure S6 shows the results of tests for overfitting in the calibration of DNN models that classify ECGs. For each training curve, the top plot shows how the value of the binary cross-entropy function minimizes with the training epoch, and the bottom plot shows consequential maximization of the model accuracy. In the DNN training curve, the first criterion of overfitting (outlined in the methods section) is met by the convergence between training (blue) and testing (orange) curves. This confirms the absence of overfitting and underfitting of the training and testing subsets used in the DNN weight optimization procedure. We satisfy a second criterion for overfitting by a combination of LOOCV for independent holdout testing as well as 7–fold cross-validation to reduce training and testing subgroup biases. We apply a third test for overfitting by assessing the performance of a DNN trained by a diagnosis vector that is randomly permuted to remove the ECG-diagnosis correlation. The training curves for each y-permuted dataset in Figure S6 show a minimization of the binary cross-entropy function (2); however, the overall accuracy of the DNN remains stagnant at about 50%

in both the training and testing curves (bottom). This indicates that the randomly calibrated DNN has a prediction accuracy akin to a 50:50 coin-flip. The *y*-permuted ROC curves in Figure S6 show the LOOCV results of the 9-lead DNN with a randomly permuted *y*-vector. Here, the AUC-ROC value is 0.470. The overlap of the logistic and diagonal no skill curves indicates a randomized DNN has no predictive ability for the classification of BrS.

Confidence Intervals Associated with Statistical Predictions

The parameters such as Sensitivity and Specificity in Table 1 themselves represent the confidence with which the model classifies electrocardiograms for a Brugada SCB outcome on scale from 0 to 1. Their magnitudes depend upon the decision threshold. To find the best-balanced decision threshold (0.498 in the present case), we apply the statistically optimized Youden's J Statistic (14).

The area under curve (AUC) of the receiver operating characteristic (ROC) obtained for a model such as ours gauges its accuracy for all decision thresholds. To compute AUC values, we implement DeLong's algorithm, as described in (15). In addition to calculating the AUC value, Delong's algorithm evaluates a covariance matrix to produce a standard error (SE) with the AUC value. The SE is calculated as the square root of the variance of the covariance matrix. From this error value, using a 95% confidence interval, we calculate the error bars on the AUC as [AUC-1.96SE, AUC+1.96SE].

Table 1 reports these 95% confidence intervals for the AUC values obtained from the analysis of the independent hold-out cases in the *Claris* (training) and *Mortara* (independent validation) cohorts, as well as those associated with the manual assessments of MD1 and 2. Note how this metric sets the manual assessments apart, and assigns a slightly higher uncertainty to AUC values derived from analyses of the smaller *Motara* dataset.

Representative ECG Data

This section presents representative ECG data obtained for patients diagnosed with Brugada Syndrome, either as manifested in a spontaneous type 1 ST-elevation in a baseline ECG or suggested by a positive type 1 response to the administration of ajmaline, as well as subjects diagnosed as healthy controls. Figures S7 through S13 show conventional 12-lead electrocardiograms with high precordial lead placements for V1-V6 (V1 II ICS, V2 II ICS, V1 III ICS, V2 III ICS, V1 IV ICS and V2 IV ICS respectively) and standard placements for leads I, II, III, aVR, aVF, aVL. These figures display traces before and after the administration of ajmaline for all cases except those diagnosed as BrS(+) on the basis of a spontaneous type 1 feature in the baseline ECG. The individual traces in blue show average representations of the ECG derived from 9 leads after trace processing as outlined in the Methods section. Each trace describes a complete depolarization-repolarization cycle, showing the characteristic ECG morphology in each lead before and during the ajmaline challenge. Importantly, these contain key diagnostic information from the full-length ECG, but are significantly smaller in size, making them far more useful for neural network processing.

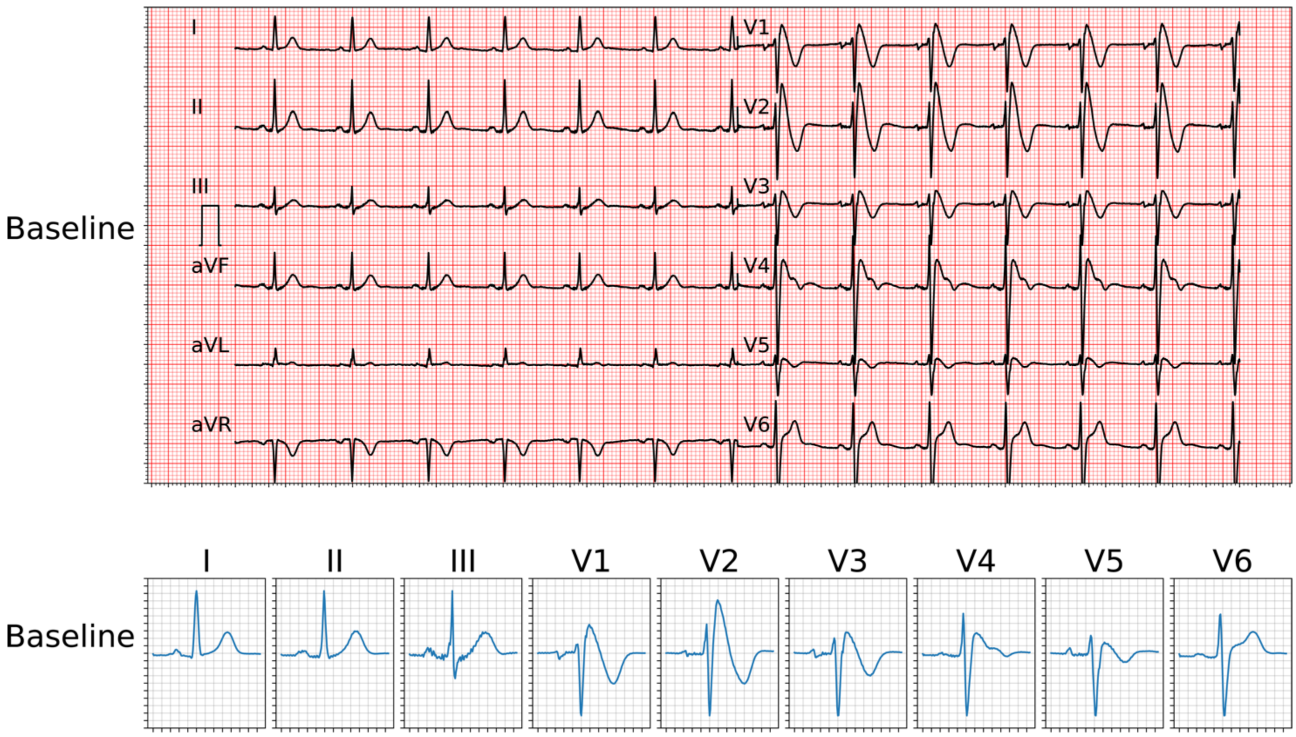

Figure S7. Subject 1233, male, survived cardiac arrest. No reported family history of BrS or SCD. (2) Raw ECG traces for a BrS(+), presenting the ST-elevation characteristic of spontaneous type 1 diagnosis in the absence of ajmaline. (bottom) ECG traces showing single average representative heartbeats measured for this patient at nine unique lead positions, without ajmaline administration. Patients in this class do not receive ajmaline. The deep neural network assigned a DNN score of $\hat{y} = 0.978 \pm 0.008$.

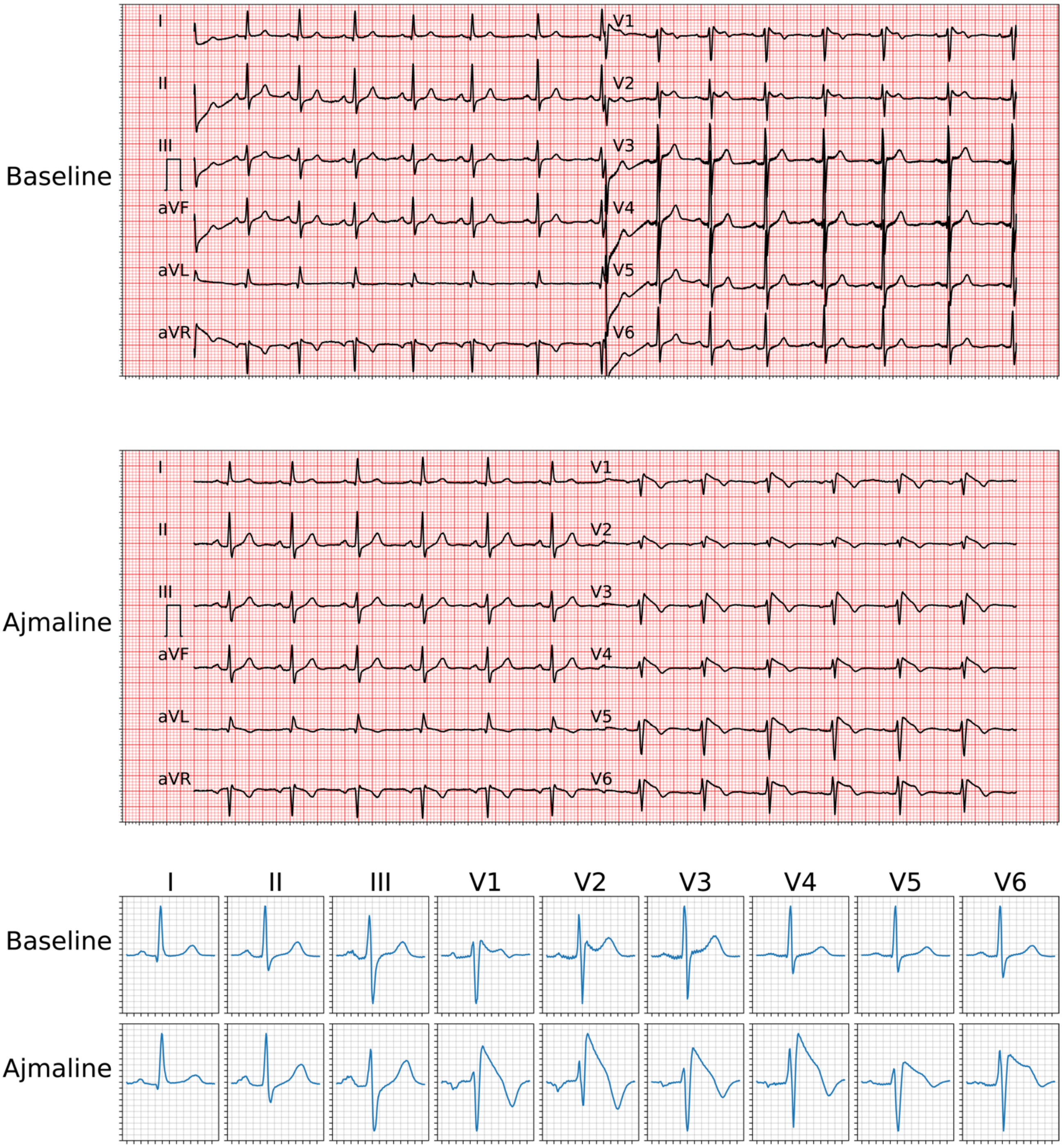

Figure S8. Subject 274, male, with a family history of SCD (brother). Here, we see a Type 2 pattern in the Baseline ECG converting to type 1 pattern after ajmaline test, confirming the BrS(+) diagnosis. (2) Raw ECG traces classified by the DNN as BrS(+), before the administration of ajmaline. (center) Raw ECG traces for the same patient after administration of ajmaline. (bottom) ECG traces showing single average representative heartbeats measured for this patient at nine unique lead positions, before ajmaline administration and after ajmaline administration. The deep neural network assigned a DNN score $\hat{y} = 0.933 \pm 0.017$.

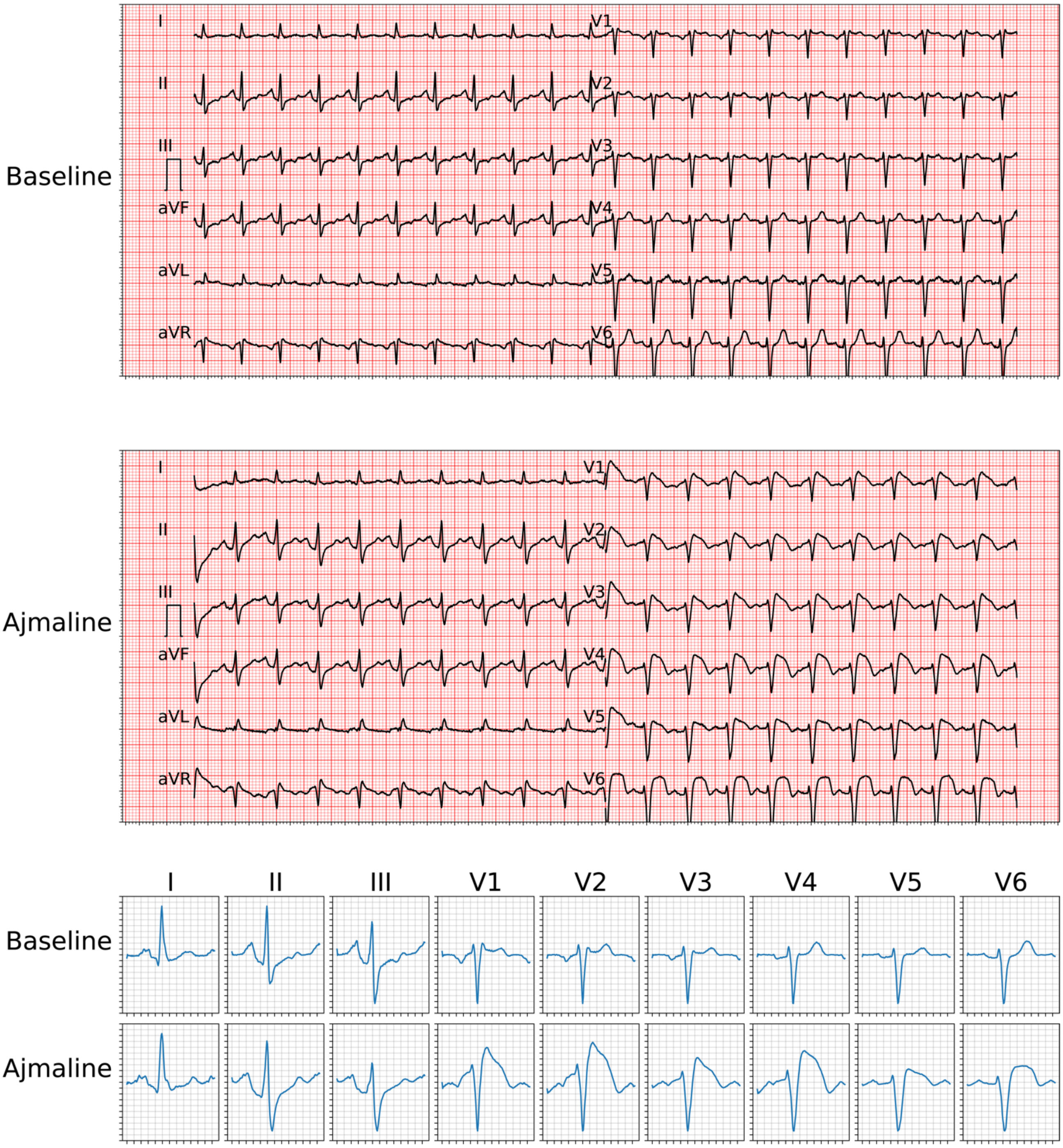

Figure S9. Subject 851, male, affected by atrial fibrillation and suspicious ECG pattern associated with a life-threatening ventricular arrhythmia during flecainide therapy. (2) Raw ECG traces forming a suspicious ECG pattern classified by the DNN as BrS(+), before the administration of ajmaline. (center) Raw ECG traces for the same patient after administration of ajmaline, revealing a type 1 pattern. (bottom) ECG traces showing single average representative heartbeats measured for this patient at nine unique lead positions, before ajmaline administration and after ajmaline administration. The deep neural network assigned a DNN score of $\hat{y} = 0.773 \pm 0.063$.

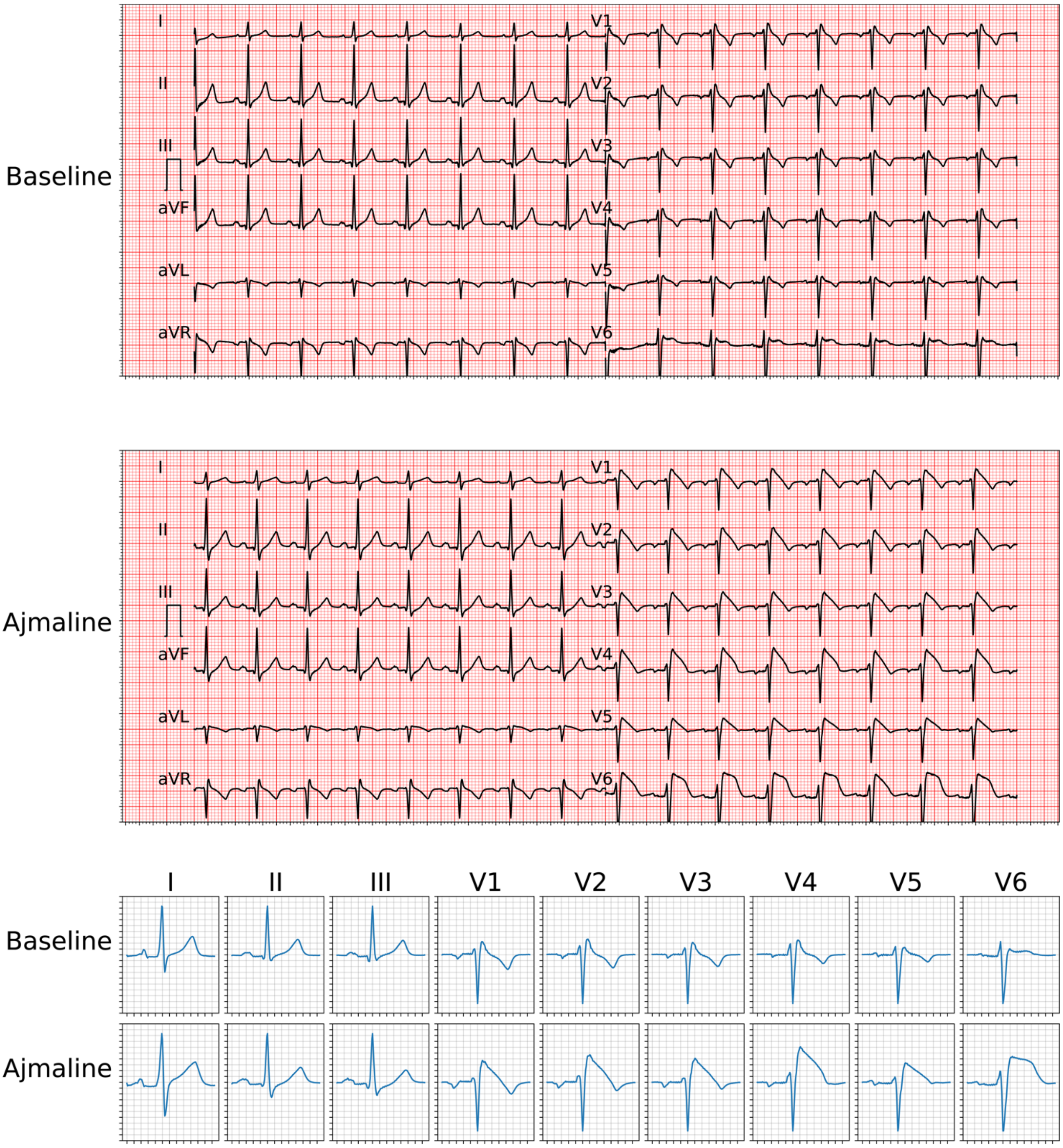

Figure S10. Subject 139, male, survived cardiac arrest with a family history of SCD (grandfather and two uncles). (2) Raw ECG traces showing a suspicious pattern with elevated J point in the high right precordial leads recorded in from the 2nd to 4th intercostal space (from V1 to V6). (center) Raw ECG traces for the same patient after administration of ajmaline showing the occurrence of a type 1 BrS pattern. (bottom) ECG traces showing single average representative heartbeats measured for this patient at nine unique lead positions, before and after ajmaline administration. The deep neural network assigned a DNN scores of $\hat{y} = 0.932 \pm 0.033$.

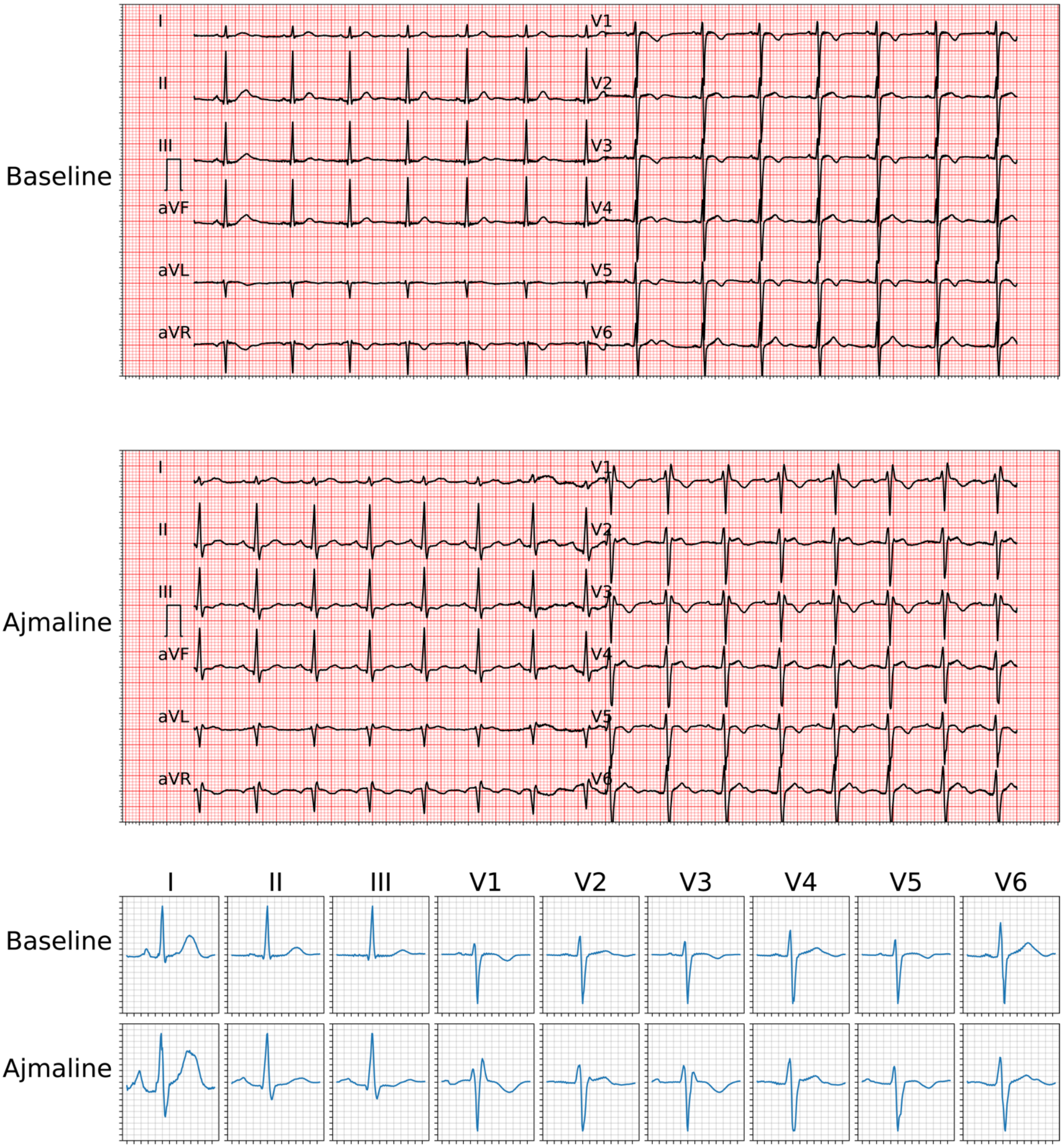

Figure S11. Subject 1111, female, with a family history of BrS and SCD. The baseline normal ECG pattern did not convert to type 1 after ajmaline challenge, thus excluding a BrS diagnosis. (2) Raw ECG traces classified by the DNN as BrS(-), before the administration of ajmaline. (center) Raw ECG traces for the same patient after administration of ajmaline. (bottom) ECG traces showing single average representative heartbeats measured for this patient at nine unique lead positions, before ajmaline administration and after ajmaline administration. The deep neural network assigned a DNN score of $\hat{y} = 0.221 \pm 0.031$.

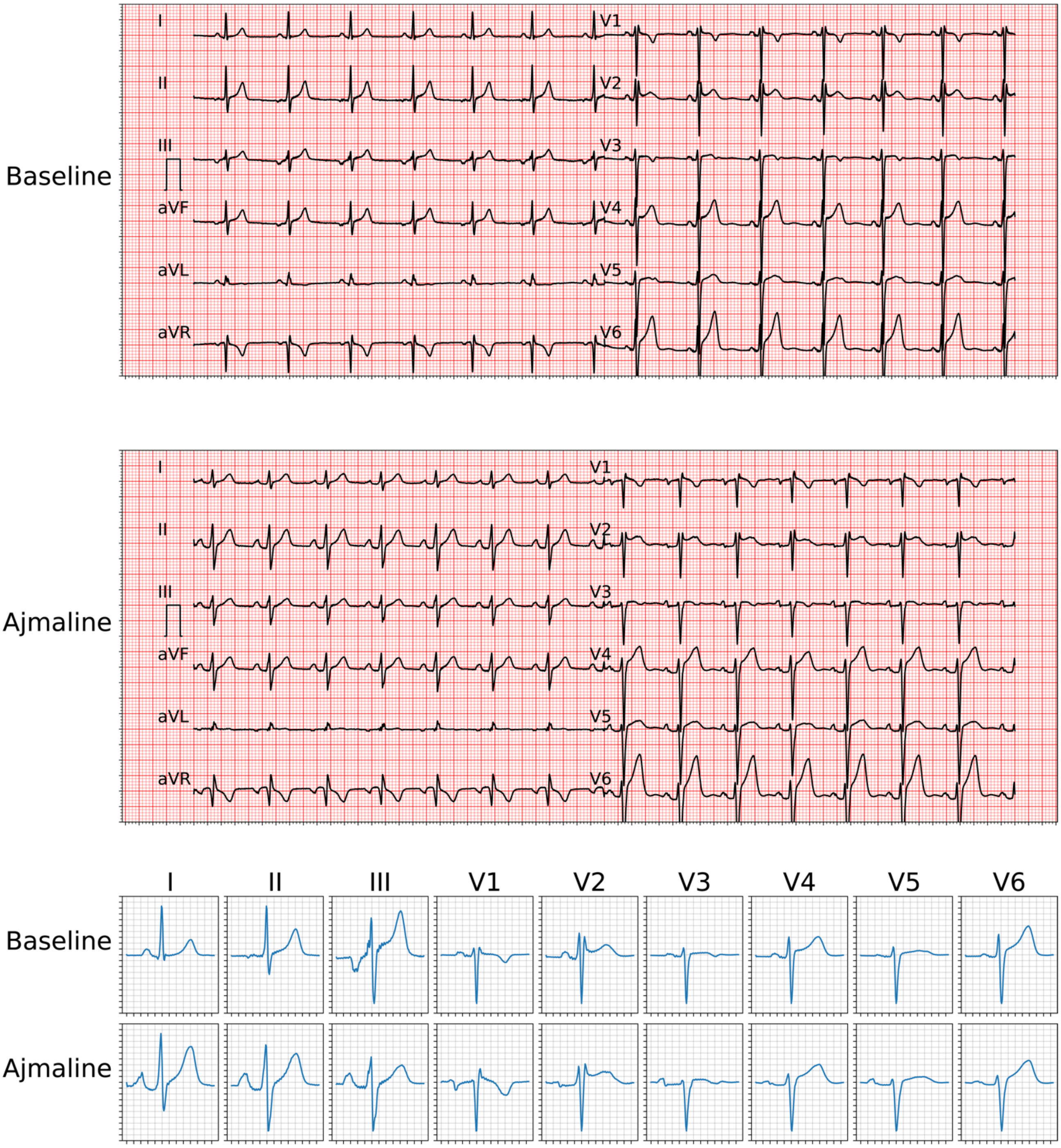

Figure S12. Suject 169, female, reported syncope during fever. Family history of BrS (mother affected with a pathogenic variant in the SCN5A mutation, and brother with SCD). She proved negative for SCN5A variants. (2) Raw ECG traces classified by the DNN as BrS(-), before the administration of ajmaline, despite a suspicious Type 2 baseline ECG pattern in the V2 II intercostal space. (center) Raw ECG traces for the same patient after administration of ajmaline, confirming a negative diagnosis. (bottom) ECG traces showing single average representative heartbeats measured for this patient at nine unique lead positions, before ajmaline administration and after ajmaline administration. The deep neural network assigned a DNN scores of $\hat{y} = 0.304 \pm 0.132$.

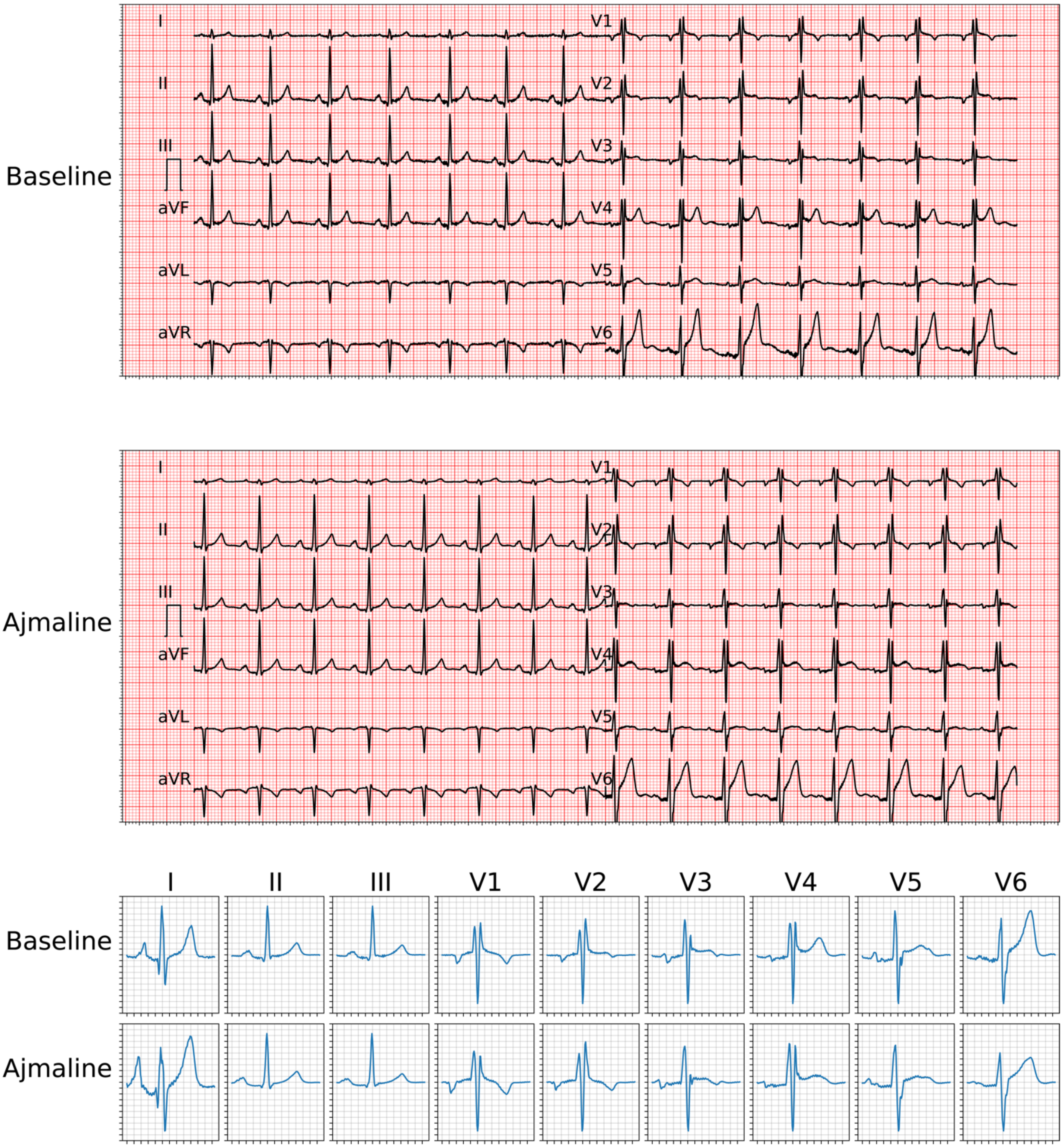

Figure S13. Subject 466, male, previous syncope and family history of SCD (father and cousin) and BrS (brother). The patient showed a suspicious incomplete right bundle branch block in high right precordial leads, which did not convert to a type 1 BrS pattern after ajmaline challenge. (2) Raw ECG traces classified by the DNN as BrS(-), before the administration of ajmaline. (center) Raw ECG traces for the same patient after administration of ajmaline. (bottom) ECG traces showing single average representative heartbeats measured for this patient at nine unique lead positions, before ajmaline administration and after ajmaline administration. The deep neural network assigned a DNN score of $\hat{y}$ = 0.251 ± 0.110.

Leave One Out Cross Validation (LOOCV) and Ensemble Learning

The most critical feature of any DNN classifier is the ability to make accurate predictions on previously unseen data. Generalization performance, which describes how well a classification algorithm works on a new dataset, is crucial to measure the real-world performance of the DNN. The present work uses cross-validation as a data subsampling technique to assess the generalization performance of our DNN classification algorithm.

The computational resources required for LOOCV exceed practicable bounds for datasets of more than a few thousand observations. This computational expense normally confines external validation practice to a single step of cross validation having withheld a fraction of observations. A dataset of fewer than 2,000 subjects present the opportunity to perform LOOCV using less than one week of computer time.

For each subject in the LOOCV process, we use ensemble learning to calculate a final classification score ($\hat{y}$). Ensemble learning, which averages the results of multiple DNN models to produce a single score, is a common practice for improving the performance of a classification algorithm. It reduces the likelihood of a misclassification due to a single poor DNN. Figure S14 depicts this cross validation and ensemble learning strategy.

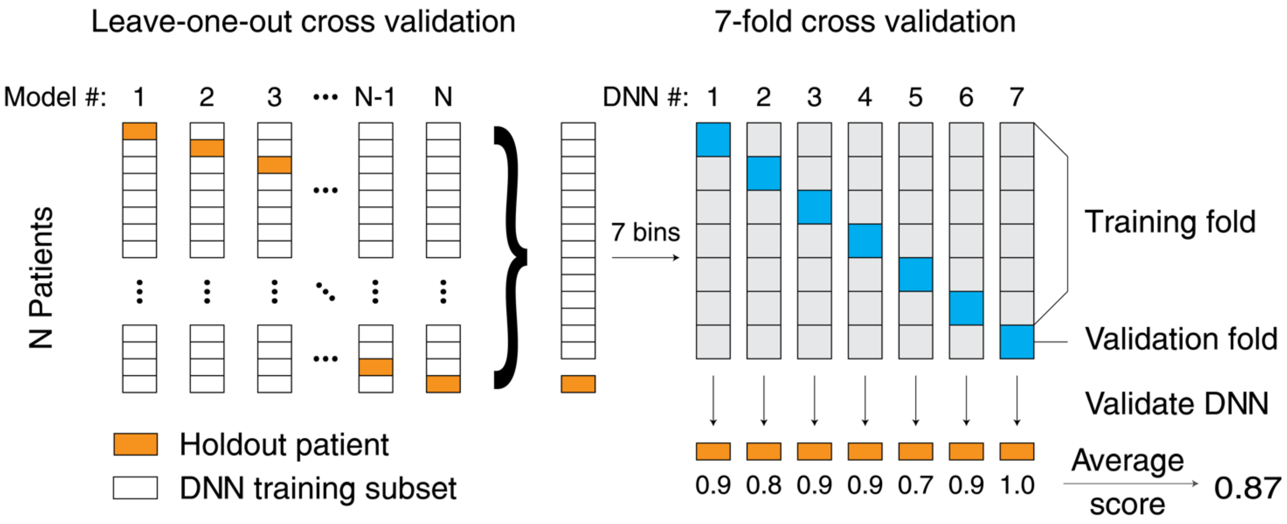

Figure S14. Data partitioning strategy for training a neural network. The overall data partitioning scheme implements leave-one-out cross-validation (LOOCV) to assess the performance of a DNN for each subject. For each subject, 7–fold cross-validation is used to separate the dataset into seven training and validation subsets, each tested on the holdout, in an effort to reduce overfitting and improve generalization. This analysis yields an average score and standard deviation for each subject across the 7–fold DNNs.

DNN Feature Importance in the 12 Lead ECG

This section presents the feature importance of the DNN. Figure S15 presents a heat map, generated by overlaying the features of greatest significance as determined by the DNN input weights, on top of a 12-lead representative ECG.

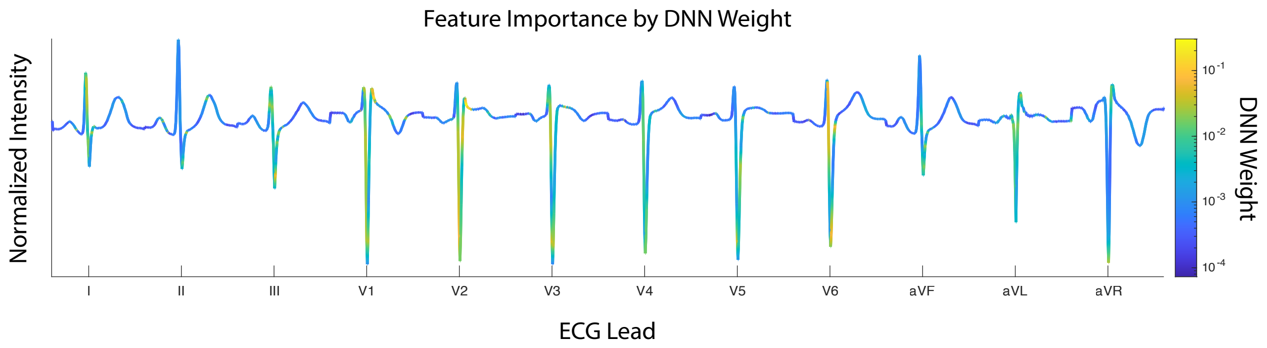

Figure S15. DNN Feature Importance**.** 12-lead representative ECG trace which is used as an input to the DNN for each subject. The colormap overlays the total sum of the DNN input weights for each segment of the representative ECG trace on a log scale. The minimum and maximum weights range from 0.0001 to 0.3021.

Understanding where a DNN places most importance in the input data is crucial for interpreting the model's decisions and for understanding its strengths and weaknesses. By identifying which ECG features the DNN uses to make predictions, we can understand which aspects of the ECG signal play the greatest role in identifying the BrS phenotype. This information can guide in the selection of new data for training, to improve the interpretability of the model and to understand how the model might fail in identifying the BrS phenotype. Additionally, by understanding where a DNN places importance on ECG features, we can also identify any potential biases in the training data that the model may have learned.

We have analyzed our DNN to determine the most significant weights of feature performance. The BrS phenotype is most characterized by the type 1 and 2 patterns. The type 1 pattern is characterized by a 'coved type' ST-segment while the type 2 pattern is characterized by a 'saddle-back type' with ST increase of $\geq$ 1mm in leads V1 to V3. A DNN classification model that aims to distinguish between these distinct type 1 and 2 patterns would likely place a significant emphasis solely on the ST-segment of leads V1 to V3. However, while the DNN does assign some importance to these regions, it also considers, but not limited to, more complex and multi-faceted features such as a non-linear combination of the ST-segments from V1 and V2, the QRS complexes in leads I, III, V1, V2, V6, and aVL. Importantly, the learning algorithm was not provided with any type of ECG classification labeling, therefore it formed its own representation of the important features indicative of a positive response to the SCB challenge. It is also important to point out that the DNN puts no importance on the 11 areas where the representative beats from each lead are concatenated together end to end.

Details in the Prediction of Brugada Syndrome for the *Claris* Training Cohort

	Overall (n = 1,455)	BrS Group (n = 758)	Control Group (n = 697)	*p* -value
**Male, n (%)**	915 (62.9)	509 (67.1)	406 (58.3)	<0.001
**Age (years) (μ ± σ)**	36.4 ± 14.9	40 ± 13.4	32.5 ± 15.4	<0.001
**Spontaneous type 1 pattern, n (%)**	103 (7.3)	103 (14)	0	<0.001
**Family history of SD, n (%)**	569 (39.1)	267 (35.2)	302 (43.4)	0.001
**Family history of BrS, n (%)**	726 (49.9)	317 (41.8)	409 (58.8)	<0.001
**Aborted Cardiac Arrest, n (%)**	23 (1.6)	14 (1.8)	9 (1.3)	0.410
**Syncope, n (%)**	286 (19.6)	159 (21)	127 (18.2)	0.209
**Asymptomatic, n (%)**	1,146 (78.8)	585 (77.2)	561 (80.5)	0.124
**SCN5A mutation, n (%)***	78 (18.4)	73 (19.2)	5 (11.6%)	<0.001
**Previous Atrial Tachyarrhythmias**				
Atrial Fibrillation, n (%)	66 (4.5)	52 (6.9)	14 (2)	<0.001
Atrial Flutter, n (%)	8 (0.5)	7 (0.9)	1 (0.1)	0.071
**Previous AVNRT, n (%)**	94 (6.6)	86 (11.3)	8 (1.1)	<0.001
**ECG characteristics**				
**Type 1 pattern**	106 (7.3)	106 (14)	0	<0.001
**Suspicious ECG**	727 (50)	396 (52.5)	331 (47.5)	0.074
Type 2 and 3 pattern	137 (9.4)	98 (12.9)	39 (5.6)	<0.001
Incomplete RBBB	561 (38.6)	276 (36.4)	285 (40.9)	0.084
RBBB	29 (2)	22 (2.9)	7 (1)	0.013
**Normal ECG waveform**	622 (42.7)	256 (33.8)	366 (52.5)	<0.001
**Comorbidities**				
Obesity, n (%)	28 (1.9)	16 (2.1)	12 (1.7)	0.703
Hypertension, n (%)	120 (8.2)	66 (8.7)	54 (7.7)	0.567
Diabetes Mellitus, n (%)	16 (1.1)	10 (1.3)	6 (0.9)	0.458
Dyslipidemia, n (%)	72 (4.9)	47 (6.2)	25 (3.6)	0.021
Thyroid disfunction, n (%)	53 (3.6)	32 (4.2)	21 (3)	0.262
Coronary Artery Disease, n (%)	2 (0.1)	0	2 (0.3)	0.229
Kidney Disease, n (%)	7 (0.5)	5 (0.7)	2 (0.3)	0.454
COPD, n (%)	4 (0.3)	3 (0.4)	1 (0.1)	0.625

Table S1. Clinical characteristics of the Claris (training) cohort.

*The genetic test result was available in 380 subjects in the BrS Group and in 43 subjects in the Control Group at the time of the analysis.

Details in the Prediction of Brugada Syndrome for the *Mortara* Validation Cohort

	Overall (n = 405)	BrS Group (n = 154)	Control Group (n = 251)	p -value
Male, n (%)	248 (61.2)	101 (65.6)	148 (58.9)	0.207
Age (years) (μ ± σ)	39±14.6	42.8±13.6	36.7±14.6	**<0.001**
Spontaneous type 1 pattern, n (%)	27 (6.7)	27 (17.5)	0	**<0.001**
Family history of SD, n (%)	179 (44.2)	69 (44.8)	110 (43.8)	0.917
Family history of BrS, n (%)	177 (43.7)	55 (35.7)	122 (48.6)	**0.013**
Aborted Cardiac Arrest, n (%)	7 (1.7)	2 (1.3)	5 (2)	0.714
Syncope, n (%)	177 (43.7)	70 (45.5)	107 (42.6)	0.607
Asymptomatic, n (%)	221 (54.6)	81 (52.6)	140 (55.8)	0.539
SCN5A mutation, n (%)*	5 (15.2)	4 (15.4)	1 (14.3)	0.072
Previous Atrial Tachyarrhythmias				
Atrial Fibrillation, n (%)	22 (5.4)	11 (7.1)	11 (4.4)	0.263
Atrial Flutter, n (%)	2 (0.5)	0	2 (0.8)	0.528
Previous AVNRT, n (%)	27 (6.7)	10 (6.5)	17 (6.7)	1
ECG characteristics				
Type 1 pattern	27 (6.7)	27 (17.5)	0	**<0.001**
Suspicious ECG	131 (32.3)	46 (28.9)	85 (33.9)	0.444
Type 2 and 3 pattern	82 (14.6)	31 (20.1)	51 (20.3)	1
Incomplete RBBB	38 (20.7)	10 (6.5)	28 (11.2)	0.160
RBBB	11 (2.7)	5 (3.2)	6 (2.4)	0.755
Normal ECG waveform	247 (61)	81 (52.6)	166 (66.1)	**0.008**
Comorbidities				
Obesity, n (%)	9 (2.2)	3 (1.9)	6 (2.4)	1
Hypertension, n (%)	51 (12.6)	23 (14.9)	28 (2)	0.282
Diabetes Mellitus, n (%)	10 (2.5)	4 (2.6)	6 (2.4)	1
Dyslipidaemia, n (%)	26 (6.4)	14 (1)	12 (4.8)	0.097
Thyroid disfunction, n (%)	16 (4)	10 (6.5)	6 (2.4)	0.063
Coronary Artery Disease, n (%)	2 (0.5)	0	2 (0.8)	0.528
Kidney Disease, n (%)	1 (0.2)	0	1 (0.4)	1
COPD, n (%)	1 (0.2)	1 (0.6)	0	0.380

Table S2. Clinical characteristics of the *Mortara* (validation) cohort. The genetic test result was available in 26 subjects in BrS Group and in 7 subjects in Control Group at the time of the analysis.

Demographics of the Training and Validation Cohorts

Subject Subgroup	Claris (training) Cohort Total [BrS - Ctl]	Mortara (validation) Cohort Total [BrS - Ctl]
All	1,154 - [596 | 558]	370 [137 - 233]
Type 1	103 - [103 | 0]	27 [27 - 0]
Type 2	75 - [74 | 1]	14 [13 - 1]
Other	957 - [400 | 557]	329 [97 - 232]
M	736 - [412 | 324]	229 [94 - 135]
F	418 - [184 | 234]	141 [43 - 98]
< 20	189 - [31 | 158]	41 [5 - 36]
20-30	248 - [102 | 146]	84 [24 - 60]
30-40	252 - [156 | 96]	82 [35 - 47]
40-50	232 - [159 | 73]	70 [36 - 34]
50-60	168 - [111 | 57]	69 [25 - 44]
> 60	65 - [37 | 28]	24 [12 - 12]

Table S3. Demographics for the ECG dataset after ECG quality thresholding factored according to ECG type, sex, and age. The *Claris* cohort contains 1,154 subjects, while there are a total of 370 exclusive subjects in the *Mortara* cohort.

The ajmaline SCB challenge presents a finite risk of death. The extensive experience of the San Donato BrS screening program, and the standby availability of a life-support team with a capacity for extracorporeal membrane oxygenation (ECMO) minimizes this risk (16). Nevertheless, as noted in the text, the occurrence of potentially life threatening arrhythmias is well documented in the literature, and the fact that the risk is low does not translate to mean zero risk. The San Donato centre has experienced a major arrhythmic event rate of <0.05% without any deaths.

Lead Subset	Sensitivity	Specificity	PPV	NPV	Accuracy	AUC-ROC	p-value
12-lead	83.1 % (495 of 596)	83.5 % (466 of 558)	84.3 % (495 of 587)	82.2 % (466 of 567)	83.3 % (961 of 1154)	0.909 ± 0.017	0.653
9-lead	85.4 % (509 of 596)	84.1 % (469 of 558)	85.1 % (509 of 598)	84.4 % (469 of 556)	84.7 % (978 of 1154)	0.913 ± 0.017	-
8-lead	80.2 % (478 of 596)	75.4 % (421 of 558)	77.7 % (478 of 615)	78.1 % (421 of 539)	77.9 % (899 of 1154)	0.850 ± 0.022	**< 0.001**
V1 + V2 II ICS	79.2 % (472 of 596)	88.7 % (495 of 558)	88.2 % (472 of 535)	80.0 % (495 of 619)	83.8 % (967 of 1154)	0.899 ± 0.018	0.910
I	76.5 % (456 of 596)	46.2 % (258 of 558)	60.3 % (456 of 756)	64.8 % (258 of 398)	61.9 % (714 of 1154)	0.683 ± 0.030	**< 0.001**
II	78.5 % (468 of 596)	62.4 % (348 of 558)	69.0 % (468 of 678)	73.1 % (348 of 476)	70.7 % (816 of 1154)	0.782 ± 0.026	**< 0.001**
III	77.3 % (461 of 596)	63.1 % (352 of 558)	69.1 % (461 of 667)	72.3 % (352 of 487)	70.5 % (813 of 1154)	0.775 ± 0.026	**< 0.001**
V1 II ICS	77.7 % (463 of 596)	84.8 % (473 of 558)	84.5 % (463 of 548)	78.1 % (473 of 606)	81.1 % (936 of 1154)	0.882 ± 0.019	0.070
V2 II ICS	74.8 % (446 of 596)	88.7 % (495 of 558)	87.6 % (446 of 509)	76.7 % (495 of 645)	81.5 % (941 of 1154)	0.876 ± 0.020	0.123
V1 III ICS	66.3 % (395 of 596)	84.2 % (470 of 558)	81.8 % (395 of 483)	70.0 % (470 of 671)	75.0 % (865 of 1154)	0.833 ± 0.023	**< 0.001**
V2 III ICS	63.8 % (380 of 596)	89.2 % (498 of 558)	86.4 % (380 of 440)	69.7 % (498 of 714)	76.1 % (878 of 1154)	0.833 ± 0.023	**< 0.001**
V1 IV ICS	65.8 % (392 of 596)	76.3 % (426 of 558)	74.8 % (392 of 524)	67.6 % (426 of 630)	70.9 % (818 of 1154)	0.775 ± 0.027	**< 0.001**
V2 IV ICs	56.0 % (334 of 596)	77.1 % (430 of 558)	72.3 % (334 of 462)	62.1 % (430 of 692)	66.2 % (764 of 1154)	0.729 ± 0.029	**< 0.001**
aVF	77.9 % (464 of 596)	64.5 % (360 of 558)	70.1 % (464 of 662)	73.2 % (360 of 492)	71.4 % (824 of 1154)	0.787 ± 0.026	**< 0.001**
aVL	70.5 % (420 of 596)	64.5 % (360 of 558)	68.0 % (420 of 618)	67.2 % (360 of 536)	67.6 % (780 of 1154)	0.734 ± 0.029	**< 0.001**
aVR	76.7 % (457 of 596)	65.2 % (364 of 558)	70.2 % (457 of 651)	72.4 % (364 of 503)	71.1 % (821 of 1154)	0.778 ± 0.027	**< 0.001**

Table S4. Results of the trained neural networks on independent holdout cases. The results are presented for 1,154 subjects through LOOCV and seven–fold cross validation of training and validation data partitions. *p*-values are calculated by a two-sided χ^2 test between the 9-lead DNN and other lead accuracy scores. A 95% confidence interval for AUC-ROC values are calculated using Delong's method.

Details of the Classification of Brugada Syndrome

Table S5 details the prediction accuracy of the 9-lead DNN regression model by ECG morphology, age, and sex. Among these factors, the prediction accuracy varies to a statistically significant degree only in the normal and suspicious ECG morphology subgroups (78.6% (367 of 467) and 87.0% (508 of 584), respectively). However, diagnostic accuracy in the type 1 patient subgroup, 100% (103 of 103), vastly exceeds that in the normal subgroup with absolute certainty.

Subject Subgroup	Sensitivity	Specificity	PPV	NPV	Accuracy	AUC-ROC	p-value
All	85.4 % (509 of 596)	84.1 % (469 of 558)	85.1 % (509 of 598)	84.4 % (469 of 556)	84.7 % (978 of 1,154)	0.913 ± 0.017	-
Type 1	100.0 % (103 of 103)				100.0 % (103 of 103)		**< 0.001**
Type 2	97.3 % (72 of 74)	0.0 % (0 of 1)	98.6 % (72 of 73)	0.0 % (0 of 2)	96.0 % (72 of 75)	0.959 ± nan	0.005
Other	79.7 % (334 of 419)	82.6 % (460 of 557)	77.5 % (334 of 431)	84.4 % (460 of 545)	81.4 % (794 of 976)	0.882 ± 0.022	0.111
M	88.1 % (363 of 412)	78.4 % (254 of 324)	83.8 % (363 of 433)	83.8 % (254 of 303)	83.8 % (617 of 736)	0.914 ± 0.020	-
F	79.3 % (146 of 184)	91.9 % (215 of 234)	88.5 % (146 of 165)	85.0 % (215 of 253)	86.4 % (361 of 418)	0.902 ± 0.032	0.250
< 20	58.1 % (18 of 31)	88.6 % (140 of 158)	50.0 % (18 of 36)	91.5 % (140 of 153)	83.6 % (158 of 189)	0.777 ± 0.105	-
20-30	80.4 % (82 of 102)	85.6 % (125 of 146)	79.6 % (82 of 103)	86.2 % (125 of 145)	83.5 % (207 of 248)	0.895 ± 0.041	0.971
30-40	88.5 % (138 of 156)	77.1 % (74 of 96)	86.2 % (138 of 160)	80.4 % (74 of 92)	84.1 % (212 of 252)	0.905 ± 0.036	0.881
40-50	88.1 % (140 of 159)	84.9 % (62 of 73)	92.7 % (140 of 151)	76.5 % (62 of 81)	87.1 % (202 of 232)	0.917 ± 0.037	0.314
50-60	88.3 % (98 of 111)	82.5 % (47 of 57)	90.7 % (98 of 108)	78.3 % (47 of 60)	86.3 % (145 of 168)	0.945 ± 0.032	0.475
> 60	89.2 % (33 of 37)	75.0 % (21 of 28)	82.5 % (33 of 40)	84.0 % (21 of 25)	83.1 % (54 of 65)	0.898 ± 0.078	0.922

Table S5. Results of the best performing DNN broken down by subject sub-clusters. The 9-lead DNN is the best performing network for the training cohort gauged by its AUC value. p-values are calculated by a χ^2 test comparing accuracy within the ECG type (Normal / Suspicious), sex, and age cohorts. A 95% confidence interval for AUC-ROC values are calculated using Delong's method.

Consideration of Additional Factors of Variation

Clinical factors of variation like age, sex, and history of cardiac events (personal history of syncope and cardiac arrest, family history of sudden death and BrS) provide a frame of reference within which cardiologists make final decisions about diagnoses and treatment options for BrS patients. Table S6 describes the effect of appending these factors of variation on classification success for the best performing 9-lead ECG dataset. We explored many permutations combining the ECG matrix with clinical factors of variation, including age, sex, and history independently, as well as the combination of age and sex, plus the combination of all three. In all cases, the addition of clinical factors of variation produced no statistical improvement to the analysis of the 9-lead ECG alone with $p > 0.05$. The consideration of additional clinical data often aids cardiologists in their diagnosis process; however, Table S6 shows little effect on the prediction accuracy of the DNN model.

Subject Subgroup	Sensitivity	Specificity	PPV	NPV	Accuracy	AUC-ROC	p-value
9-lead (ECG only)	85.4 % (509 of 596)	84.1 % (469 of 558)	85.1 % (509 of 598)	84.4 % (469 of 556)	84.7 % (978 of 1154)	0.913 ± 0.017	-
ECG + Age	85.4 % (509 of 596)	83.0 % (463 of 558)	84.3 % (509 of 604)	84.2 % (463 of 550)	84.2 % (972 of 1154)	0.913 ± 0.016	0.864
ECG + Sex	84.9 % (506 of 596)	82.6 % (461 of 558)	83.9 % (506 of 603)	83.7 % (461 of 551)	83.8 % (967 of 1154)	0.912 ± 0.017	0.910
ECG + History	85.2 % (508 of 596)	82.8 % (462 of 558)	84.1 % (508 of 604)	84.0 % (462 of 550)	84.1 % (970 of 1154)	0.911 ± 0.017	0.955
ECG + Age + Sex	85.6 % (510 of 596)	82.8 % (462 of 558)	84.2 % (510 of 606)	84.3 % (462 of 548)	84.2 % (972 of 1154)	0.910 ± 0.017	0.864
ECG + Age + Sex + History	85.2 % (508 of 596)	83.2 % (464 of 558)	84.4 % (508 of 602)	84.1 % (464 of 552)	84.2 % (972 of 1154)	0.906 ± 0.017	0.864

Table S6. Results of the trained neural networks on independent holdout cases with various combinations of factors of variation added into the dataset. Factors of variation include age, sex, and personal and family history of heart conditions (syncope, history of cardiac arrest, family history of sudden death and family history of BrS). A 9-lead dataset (all except the unipolar limb leads) is used as the ECG matrix, as it is the best performing of all lead permutations explored. The addition of clinical subject data often aids physicians in their diagnosis process, although here it is shown to have little effect on the decision making of the DNN. p-values are calculated by a two-sided $\chi 2$ test between the ECG-only and factors of variation accuracy scores. A 95% confidence interval for AUC-ROC values are calculated using Delong's method.

Table S7 details the prediction accuracy of the 9-lead DNN model by ECG morphology, age, and sex when applied to the *Mortara* independent validation cohort. All subgroups classify statistically similar (p-value > 0.05) to the main subgroup of comparison.

Subject Subgroup	Sensitivity	Specificity	PPV	NPV	Accuracy	AUC-ROC	p-value
All	79.6 % (109 of 137)	93.6 % (218 of 233)	87.9 % (109 of 124)	88.6 % (218 of 246)	88.4 % (327 of 370)	0.934 ± 0.027	-
Type 1	100.0 % (27 of 27)	-	100.0 % (27 of 27)	-	100.0 % (27 of 27)		0.061
Type 2	100.0 % (13 of 13)	0.0 % (0 of 1)	92.9 % (13 of 14)	-	92.9 % (13 of 14)	0.692 ± nan	0.606
Other	71.1 % (69 of 97)	94.0 % (218 of 232)	83.1 % (69 of 83)	88.6 % (218 of 246)	87.2 % (287 of 329)	0.911 ± 0.036	0.644
M	85.1 % (80 of 94)	91.9 % (124 of 135)	87.9 % (80 of 91)	89.9 % (124 of 138)	89.1 % (204 of 229)	0.926 ± 0.037	-
F	67.4 % (29 of 43)	95.9 % (94 of 98)	87.9 % (29 of 33)	87.0 % (94 of 108)	87.2 % (123 of 141)	0.946 ± 0.039	0.590
< 20	40.0 % (2 of 5)	100.0 % (36 of 36)	100.0 % (2 of 2)	92.3 % (36 of 39)	92.7 % (38 of 41)	0.800 ± 0.242	-
20-30	79.2 % (19 of 24)	95.0 % (57 of 60)	86.4 % (19 of 22)	91.9 % (57 of 62)	90.5 % (76 of 84)	0.934 ± 0.073	0.683
30-40	82.9 % (29 of 35)	93.6 % (44 of 47)	90.6 % (29 of 32)	88.0 % (44 of 50)	89.0 % (73 of 82)	0.951 ± 0.051	0.519
40-50	75.0 % (27 of 36)	85.3 % (29 of 34)	84.4 % (27 of 32)	76.3 % (29 of 38)	80.0 % (56 of 70)	0.868 ± 0.085	0.073
50-60	84.0 % (21 of 25)	93.2 % (41 of 44)	87.5 % (21 of 24)	91.1 % (41 of 45)	89.9 % (62 of 69)	0.957 ± 0.041	0.618
> 60	91.7 % (11 of 12)	91.7 % (11 of 12)	91.7 % (11 of 12)	91.7 % (11 of 12)	91.7 % (22 of 24)	0.972 ± 0.052	0.882

Table S7. Validation of the Mortara *cohort* by the 9-lead DNN broken down by subject sub-clusters. *p*-values are calculated by a χ^2 test comparing accuracy within the ECG type (All / Type 1 / Type 2 / Other), sex, and age cohorts. A 95% confidence interval for AUC-ROC values are calculated using Delong's method.

Summary of sub-cluster correlations

Within the *Claris* dataset 73 of the 75 patients with a type 2 or 3 waveform responded positively to an ajmaline challenge, and were diagnosed BrS(+). The validation exercise just described correctly identified 72 patients as positive, for a Sensitivity of 97.3%. When applied to the entirely separate *Mortara* dataset, a completely independent set of *Claris*-trained DNN models correctly identified 13 of 13 BrS(+) patients for a sensitivity of 100%. The results thus offer little room for improvement by reclassification. Although this classification success is interesting (and gratifying), the numbers of such patients are small, and we do not wish to associate a perceived type 2 or 3 pattern with a definitive diagnosis of BrS. Indeed, a bias along these lines might have caused the BrS(+) diagnoses of MD1 to include so many apparent BrS phenocopies.

Our model-assisted protocol distinguishes potential phenocopies as patients who present ECGs classified as BrS(+) but respond negatively to an SCB challenge. In the clinical setting of San Donato BrS screening program, a complete clinical evaluation and follow up adds weight to such a distinction. By this criterion, 98 of the 607 patients in the *Claris* cohort classified by the DNN as BrS(+) are phenocopies. DNN analysis of the *Motara* cohort labels 224 patients as BrS(+), of whom 15 are recognized by clinical analysis as phenocopies. Our Supplemental Materials now points this out, and mentions that of 161 patients labeled as BrS(+) by MD1 (the expert physician) 63 were phenocopies by this definition.

Convolutional Neural Network (CNN) Processing

Our convolutional neural network (CNN) works by optimizing a set of features learned from the 9-lead ECG data. We train the CNN using the ECG data from subjects in the Claris cohort, and then validate the trained CNN with ECGs in the Mortara cohort. The raw traces are subject to the same filtering and denoising steps outlined in the Methods section. Each trace is then down-sampled from 1000 Hz to 250 Hz before input into the CNN. We determined the structure of the CNN presented in Table S8 by an exhaustive grid search of the following hyperparameters (optimized values high-lighted):

- Number of convolutional layers: 1, 2, **3**, …, 10
    - Filters: 1, 5, **10**, 16, 32, 64, 128
    - Kernel size: 4, **8**, 16, 32
    - Pool size: **2**, 3, 4
    - Pooling strides: **1**, 2, 3
- Gradient Descent Optimizers: **adam**,[13] adagrad,[9] rmsprop, sgd
- Dropout Layers[14]
- Activation / Batch Normalization Sequence
- ECG trace length (seconds): 2, **5**, 10, 20, 30
- Number of Dense layers before output layer: **1**, 2, 3, 4, 5
    - Nodes per hidden dense layer: 1, 2, 3, 4, 5, 10, **15**, 20, 50

Layer (type)	Output Shape	Number of Parameters
conv1d_1 (Conv1D)	(None, 1243, 10)	970
max_pooling1d_1 (MaxPooling1D)	(None, 621, 10)	0
conv1d_2 (Conv1D)	(None, 614, 10)	810
max_pooling1d_1 (MaxPooling1D)	(None, 307, 10)	0
conv1d_3 (Conv1D)	(None, 300, 10)	810
max_pooling1d_1 (MaxPooling1D)	(None, 150, 10)	0
flatten_1 (Flatten)	(None, 1500)	0
dense_1 (Dense)	(None, 15)	22515
gaussian_noise_1 (GaussianNoise)	(None, 15)	0
activation_1 (Activation)	(None, 15)	0
batch_normalization_1 (BatchNormalization)	(None, 15)	60
dense_2 (Dense)	(None, 1)	16
Total params: 25,181		
Trainable params: 25,151		
Non-trainable params: 30		

Table S8. CNN structure used for validation of the *Validation* cohort.

Convolutional Neural Network Validation Results

Below, Table S9 details the prediction accuracy of the 9-lead CNN model by ECG morphology, age, and sex when applied to the Mortara independent validation cohort. Among these factors, the type 1 patient subgroup with accuracy of 100% (27 of 27) vastly exceeds that in the total cohort.

Subject Subgroup	Sensitivity	Specificity	PPV	NPV	Accuracy	AUC-ROC	p-value
ALL	74.5 % (102 of 137)	88.8 % (207 of 233)	79.7 % (102 of 128)	85.5 % (207 of 242)	83.5 % (309 of 370)	0.863 ± 0.041	0.057
TYPE 1	100.0 % (27 of 27)	-	100.0 % (27 of 27)	-	100.0 % (27 of 27)	-	0.022
TYPE 2	92.3 % (12 of 13)	100.0 % (1 of 1)	100.0 % (12 of 12)	50.0 % (1 of 2)	92.9 % (13 of 14)	0.923 ± nan	0.351
OTHER	64.9 % (63 of 97)	88.8 % (206 of 232)	70.8 % (63 of 89)	85.8 % (206 of 240)	81.8 % (269 of 329)	0.821 ± 0.053	0.541
M	74.5 % (70 of 94)	88.9 % (120 of 135)	82.4 % (70 of 85)	83.3 % (120 of 144)	83.0 % (190 of 229)	0.856 ± 0.054	-
F	74.4 % (32 of 43)	88.8 % (87 of 98)	74.4 % (32 of 43)	88.8 % (87 of 98)	84.4 % (119 of 141)	0.873 ± 0.063	0.719
0-20	40.0 % (2 of 5)	97.2 % (35 of 36)	66.7 % (2 of 3)	92.1 % (35 of 38)	90.2 % (37 of 41)	0.689 ± 0.302	-
20-30	58.3 % (14 of 24)	93.3 % (56 of 60)	77.8 % (14 of 18)	84.8 % (56 of 66)	83.3 % (70 of 84)	0.810 ± 0.113	0.302
30-40	85.7 % (30 of 35)	85.1 % (40 of 47)	81.1 % (30 of 37)	88.9 % (40 of 45)	85.4 % (70 of 82)	0.900 ± 0.081	0.448
40-50	77.8 % (28 of 36)	82.4 % (28 of 34)	82.4 % (28 of 34)	77.8 % (28 of 36)	80.0 % (56 of 70)	0.842 ± 0.101	0.158
50-60	76.0 % (19 of 25)	86.4 % (38 of 44)	76.0 % (19 of 25)	86.4 % (38 of 44)	82.6 % (57 of 69)	0.894 ± 0.072	0.272
60+	75.0 % (9 of 12)	83.3 % (10 of 12)	81.8 % (9 of 11)	76.9 % (10 of 13)	79.2 % (19 of 24)	0.813 ± 0.192	0.212

Table S9. Results of the Mortara cohort validation by the 9-lead CNN broken down by subject sub-clusters. *p*-values are calculated by a χ^2 test comparing accuracy to the Mortara accuracy, sex, and age cohorts. A 95% confidence interval for AUC-ROC values are calculated using Delong's method.

The CNN methodology applied to develop the classification model for the purposes of the validation detailed above shares similarities with previous research exploring the ability of machine learning models to recognize signs of BrS in clinical ECGs. In every case, those studies trained neural networks to recognize the appearance of a coved ST elevation (type 1 BrS signature) in minimally processed ECG traces. The prediction success of these studies, as measured by accuracy (correctly classified subjects as a percent of the total validation set) ranged from 76.9% to 93.8%. The CNN model above, trained to predict the result of the BrS(+) SCB challenge in an ECG of any type, achieves an accuracy of

100% in recognizing type 1 ECGs, both by leave one out cross validation (LOOCV) in the training cohort of 103 patients, as described in the text, and by independent classification of the 27 patients in the Mortara validation cohort.

The most important distinction of our approach is the capacity we prove to identify the Brugada Syndrome phenotype in all ECGs, including the majority that are evidently asymptomatic. Unlike Liu et al. and other cited researchers, Dimitri et al., Liao et al. and Nakamura et al., the training of our DNN does not confine it to recognize BrS only when signified by a spontaneous type 1 ST-elevation.

In addition, our study differs from others in our ECG preprocessing methodology. Many machine learning (ML) applications, including those by Liu et al. and Dimitri et al., use convolutional layers to extract features from minimally processed 12-lead ECG traces. In contrast, our methodology employs a powerful preprocessing step that reduces the dimensionality of the input ECG data to a higher-fidelity single representative beat for each lead. This step improves the performance of the learning process by providing a superior representation of the ECG data, as demonstrated by the comparison of our DNN (AUC 0.934±0.027, accuracy 88.4%) with our best CNN (AUC 0.863±0.041, accuracy 83.5%). Additionally, when we apply a CNN to our representative beat instead of a minimally filtered ECG trace, we see little difference in the learning performance in our training cohort, indicating significant added value in our preprocessing.

Our method offers several advantages in terms of machine learning (ML) training and deployment. One advantage is that our additional preprocessing step has a minimal impact on the deployment time, while still providing significant benefits in terms of the dimensionality reduction. This reduction in dimensionality improves the training time for producing our final DNN models when compared to using traditional convolutional neural network (CNN) models. This distinction is beneficial as it allows us to easily retrain the models with new data and scale the use of our method to larger datasets in the future.

Our study distinguishes itself with its innovative ECG preprocessing methodology which improves the performance of the learning process by providing a superior representation of the ECG data, as well as its ability to identify patients with seemingly normal ECGs that would respond positively to a sodium channel blocker challenge, something not addressed in the aforementioned studies. The added value in our preprocessing and its distinction from Liu et al. and Dimitri et al. sets our study apart from currently published literature and adds value for researchers and physicians in the field.

Detailed Clinician Results

Below, Table S10 details the prediction accuracy of the Expert clinician (MD1) by ECG morphology, age, and sex for the Mortara cohort. Among these factors, the prediction accuracy varies to a statistically significant degree compared to the DNN overall. M.D. 1 successfully identified 100% (27 of 27) of the patients exhibiting type 1 ECG 72.4% (268 of 370) of subjects overall.

Subject Subgroup	Sensitivity	Specificity	PPV	NPV	Accuracy	AUC-ROC	p-value
ALL	71.5 % (98 of 137)	73.0 % (170 of 233)	60.9 % (98 of 161)	81.3 % (170 of 209)	72.4 % (268 of 370)	0.722 ± 0.047	4.65E-08
TYPE 1	100.0 % (27 of 27)	-	100.0 % (27 of 27)	-	100.0 % (27 of 27)	-	0.002
TYPE 2	92.3 % (12 of 13)	0.0 % (0 of 1)	92.3 % (12 of 13)	0.0 % (0 of 1)	85.7 % (12 of 14)	0.462 ± nan	0.272
OTHER	60.8 % (59 of 97)	73.3 % (170 of 232)	48.8 % (59 of 121)	81.7 % (170 of 208)	69.6 % (229 of 329)	0.671 ± 0.057	0.410
M	71.3 % (67 of 94)	73.3 % (99 of 135)	65.0 % (67 of 103)	78.6 % (99 of 126)	72.5 % (166 of 229)	0.723 ± 0.059	-
F	72.1 % (31 of 43)	72.4 % (71 of 98)	53.4 % (31 of 58)	85.5 % (71 of 83)	72.3 % (102 of 141)	0.723 ± 0.081	0.975
0-20	40.0 % (2 of 5)	83.3 % (30 of 36)	25.0 % (2 of 8)	90.9 % (30 of 33)	78.0 % (32 of 41)	0.617 ± 0.248	1
20-30	79.2 % (19 of 24)	78.3 % (47 of 60)	59.4 % (19 of 32)	90.4 % (47 of 52)	78.6 % (66 of 84)	0.787 ± 0.098	0.947
30-40	85.7 % (30 of 35)	72.3 % (34 of 47)	69.8 % (30 of 43)	87.2 % (34 of 39)	78.0 % (64 of 82)	0.790 ± 0.087	-
40-50	61.1 % (22 of 36)	55.9 % (19 of 34)	59.5 % (22 of 37)	57.6 % (19 of 33)	58.6 % (41 of 70)	0.585 ± 0.117	0.037
50-60	64.0 % (16 of 25)	72.7 % (32 of 44)	57.1 % (16 of 28)	78.0 % (32 of 41)	69.6 % (48 of 69)	0.684 ± 0.117	0.334
60+	75.0 % (9 of 12)	66.7 % (8 of 12)	69.2 % (9 of 13)	72.7 % (8 of 11)	70.8 % (17 of 24)	0.708 ± 0.189	0.515

Table S10. Results obtained in the validation of the Mortara dataset upon inspection by an Expert Clinician (M.D. 1) broken down by subject sub-clusters. *p*-values are calculated by a χ^2 test comparing accuracy to the DNN validation of the Mortara cohort. A 95% confidence interval for AUC-ROC values are calculated using Delong's method.

Below, Table S11 details the prediction accuracy of the Resident clinician (M.D. 2) by ECG morphology, age, and sex for the Mortara cohort. Among these factors, the prediction accuracy varies to a statistically significant degree compared to the DNN overall. M.D. 2 successfully identified 92.6% (27 of 27) of the patients exhibiting type 1 ECG and 58.9% (218 of 370) of subjects overall.

Subject Subgroup	Sensitivity	Specificity	PPV	NPV	Accuracy	AUC-ROC	p-value
ALL	55.5 % (76 of 137)	60.9 % (142 of 233)	45.5 % (76 of 167)	70.0 % (142 of 203)	58.9 % (218 of 370)	0.582 ± 0.052	9.41E-20
TYPE 1	92.6 % (25 of 27)	-	100.0 % (25 of 25)	0.0 % (0 of 2)	92.6 % (25 of 27)	-	0.001
TYPE 2	61.5 % (8 of 13)	100.0 % (1 of 1)	100.0 % (8 of 8)	16.7 % (1 of 6)	64.3 % (9 of 14)	0.808 ± nan	0.688
OTHER	44.3 % (43 of 97)	60.8 % (141 of 232)	32.1 % (43 of 134)	72.3 % (141 of 195)	55.9 % (184 of 329)	0.526 ± 0.059	0.424
M	60.6 % (57 of 94)	64.4 % (87 of 135)	54.3 % (57 of 105)	70.2 % (87 of 124)	62.9 % (144 of 229)	0.625 ± 0.064	-
F	44.2 % (19 of 43)	56.1 % (55 of 98)	30.6 % (19 of 62)	69.6 % (55 of 79)	52.5 % (74 of 141)	0.502 ± 0.090	0.048
0-20	40.0 % (2 of 5)	72.2 % (26 of 36)	16.7 % (2 of 12)	89.7 % (26 of 29)	68.3 % (28 of 41)	0.561 ± 0.251	-
20-30	62.5 % (15 of 24)	70.0 % (42 of 60)	45.5 % (15 of 33)	82.4 % (42 of 51)	67.9 % (57 of 84)	0.662 ± 0.115	0.961
30-40	77.1 % (27 of 35)	59.6 % (28 of 47)	58.7 % (27 of 46)	77.8 % (28 of 36)	67.1 % (55 of 82)	0.684 ± 0.100	0.892
40-50	47.2 % (17 of 36)	47.1 % (16 of 34)	48.6 % (17 of 35)	45.7 % (16 of 35)	47.1 % (33 of 70)	0.471 ± 0.119	0.031
50-60	40.0 % (10 of 25)	52.3 % (23 of 44)	32.3 % (10 of 31)	60.5 % (23 of 38)	47.8 % (33 of 69)	0.461 ± 0.123	0.037
60+	41.7 % (5 of 12)	58.3 % (7 of 12)	50.0 % (5 of 10)	50.0 % (7 of 14)	50.0 % (12 of 24)	0.500 ± 0.206	0.143

Table S11. Results obtained in the validation of the Mortara cohort upon inspection by a Resident Clinician (M.D. 2) broken down by subject sub-clusters. *p*-values are calculated by a χ^2 test comparing accuracy to the DNN validation of the Mortara cohort. A 95% confidence interval for AUC-ROC values are calculated using Delong's method.

DNN Performance Comparison with Other Popular Classifiers

Table S12 compares the performance of a DNN classifier trained on the 9-lead dataset with other popular supervised learning techniques such as decision trees, Naïve Bayes, support vector machines (SVM), and *k*-nearest neighbors (KNN), among others. The results show that DNN models outperform these 24 common classifiers by a significant margin. Only two methods offer AUC values approaching that of the DNN (0.895), Boosted Trees (0.806), and Linear Support Vector Machine (SVM) (0.802). Importantly, even though both Boosted Trees and Linear SVM classifiers yield AUC-ROC value exceeding 0.8, neither offers a sensitivity value above 69%, indicating that they are unbalanced classifiers, despite the selection of an optimal cutoff value in each case using the ROC.

Learning Method	Sensitivity	Specificity	Accuracy	AUC-ROC
Deep Neural Network	85.4	84.1	84.7	0.913 ± 0.017
Fine Tree	66.8 ± 1.5	62.7 ± 1.9	64.0 ± 1.0	0.622 ± 0.015
Medium Tree	64.7 ± 1.2	72.4 ± 1.0	68.3 ± 0.7	0.726 ± 0.005
Coarse Tree	65.7 ± 1.3	77.6 ± 2.7	71.3 ± 0.6	0.748 ± 0.004
Linear Discriminant	58.3 ± 2.5	57.9 ± 1.6	58.1 ± 1.4	0.582 ± 0.008
Logistic Regression	51.2 ± 0.5	51.1 ± 1.8	51.2 ± 0.8	0.512 ± 0.008
Gaussian Naïve Bayes	58.8 ± 0.2	77.9 ± 0.6	67.8 ± 0.4	0.710 ± 0.001
Kernel Naïve Bayes	64.8 ± 0.9	68.5 ± 0.7	66.5 ± 0.7	0.694 ± 0.004
Linear SVM	68.9 ± 0.8	78.6 ± 0.9	73.5 ± 0.5	0.802 ± 0.004
Quadratic SVM	69.1 ± 0.3	74.6 ± 1.4	71.7 ± 0.7	0.788 ± 0.004
Cubic SVM	71.5 ± 1.4	70.2 ± 1.2	70.9 ± 0.7	0.768 ± 0.004
Fine Gaussian SVM	99.5 ± 0.1	0.2 ± 0.1	52.9 ± 0.1	0.602 ± 0.008
Medium Gaussian SVM	70.7 ± 1.0	73.6 ± 1.1	72.0 ± 0.9	0.792 ± 0.004
Coarse Gaussian SVM	64.5 ± 0.9	77.1 ± 0.5	70.4 ± 0.3	0.780 ± 0.001
Fine KNN	57.9 ± 0.9	60.5 ± 1.4	59.1 ± 0.3	0.590 ± 0.001
Medium KNN	50.1 ± 1.0	79.2 ± 1.0	63.8 ± 0.4	0.698 ± 0.004
Coarse KNN	49.5 ± 0.9	83.6 ± 0.7	65.5 ± 0.3	0.738 ± 0.004
Cosine KNN	58.6 ± 1.1	71.3 ± 0.9	64.5 ± 0.3	0.714 ± 0.005
Cubic KNN	52.1 ± 0.9	77.3 ± 0.8	64.0 ± 0.4	0.692 ± 0.004
Weighted KNN	58.6 ± 1.1	69.7 ± 0.9	63.8 ± 0.4	0.696 ± 0.005
Boosted Trees	69.0 ± 0.3	78.2 ± 1.6	73.3 ± 0.7	0.806 ± 0.005
Bagged Trees	66.2 ± 2.1	74.7 ± 1.6	70.2 ± 1.7	0.770 ± 0.012
Subspace Discriminant	61.3 ± 2.0	62.1 ± 1.8	61.7 ± 1.6	0.658 ± 0.028
Subspace KNN	62.5 ± 1.0	56.9 ± 0.8	59.9 ± 0.5	0.632 ± 0.008
RUSBoosted Trees	62.9 ± 1.4	81.8 ± 0.6	71.8 ± 0.8	0.784 ± 0.005

Table S12. Results of comparing widely adopted supervised learning techniques to the DNN for the 9-lead dataset. This dataset was chosen because it has the best overall performance among the DNNs with respect to AUC value. Each result is the average accuracy of a 5-fold cross-validation over 5 independent trials.

References

1. Romero FP, Romaguera LV, Vázquez-Seisdedos CR, Costa MGF, Neto JE. Baseline wander removal methods for ECG signals: A comparative study. arXiv preprint arXiv:180711359. 2018.
2. McManus CD, Neubert K-D, Cramer E. Characterization and elimination of AC noise in electrocardiograms: a comparison of digital filtering methods. Computers and Biomedical research. 1993;26(1):48-67.
3. Hotelling H. The generalization of Student's ratio. Breakthroughs in statistics: Springer; 1992. p. 54-65.
4. Ester M, Kriegel H-P, Sander J, Xu X, editors. A density-based algorithm for discovering clusters in large spatial databases with noise. kdd; 1996.
5. Park MY, Hastie T. L1-regularization path algorithm for generalized linear models. Journal of the Royal Statistical Society: Series B (Statistical Methodology). 2007;69(4):659-77.
6. An G. The effects of adding noise during backpropagation training on a generalization performance. Neural computation. 1996;8(3):643-74.
7. Nair V, Hinton GE, editors. Rectified linear units improve restricted boltzmann machines. Icml; 2010.
8. Ioffe S, Szegedy C, editors. Batch normalization: Accelerating deep network training by reducing internal covariate shift. International conference on machine learning; 2015: PMLR.
9. Ruder S. An overview of gradient descent optimization algorithms. arXiv preprint arXiv:160904747. 2016.
10. Zadrozny B, Elkan C, editors. Transforming classifier scores into accurate multiclass probability estimates. Proceedings of the eighth ACM SIGKDD international conference on Knowledge discovery and data mining; 2002.
11. Niculescu-Mizil A, Caruana R, editors. Predicting good probabilities with supervised learning. Proceedings of the 22nd international conference on Machine learning; 2005.
12. Kingma DP, Ba JL. Adam: A method for stochastic optimization. 3rd International Conference on Learning Representations, ICLR 2015 - Conference Track Proceedings. 2015:1–15.
13. Srivastava N, Hinton G, Krizhevsky A, Sutskever I, Salakhutdinov R. Dropout: A Simple Way to Prevent Neural Networks from Overfitting. Journal of Machine Learning Research. 2014;15(56):1929-58.
14. Youden WJ. Index for rating diagnostic tests. Cancer. 1950;3(1):32-5.
15. X. Sun WX. Fast implementation of DeLong's algorithm for comparing the areas under correlated receiver operating characteristic curves. IEEE Signal Processing Letters,. 2014;21(11):1389-93.
16. Ciconte G, Monasky, M. M., Vicedomini, G., Borrelli, V., Giannelli, L., Pappone, C. . Unusual response to ajmaline test in Brugada syndrome patient leads to extracorporeal membrane oxygenator support. EP Europace. 2019;21(10):1574.
